# A Comprehensive Curation Shows the Dynamic Evolutionary Patterns of Prokaryotic CRISPRs

**DOI:** 10.1155/2016/7237053

**Published:** 2016-04-18

**Authors:** Guoqin Mai, Ruiquan Ge, Guoquan Sun, Qinghan Meng, Fengfeng Zhou

**Affiliations:** ^1^Shenzhen Institutes of Advanced Technology and Key Lab for Health Informatics, Chinese Academy of Sciences, Shenzhen, Guangdong 518055, China; ^2^Shenzhen College of Advanced Technology, University of Chinese Academy of Sciences, Shenzhen, Guangdong 518055, China; ^3^College of Computer Science and Technology, Jilin University, Changchun, Jilin 130012, China; ^4^Key Laboratory of Symbolic Computation and Knowledge Engineering of Ministry of Education, Jilin University, Changchun, Jilin 130012, China

## Abstract

*Motivation.* Clustered regularly interspaced short palindromic repeat (CRISPR) is a genetic element with active regulation roles for foreign invasive genes in the prokaryotic genomes and has been engineered to work with the CRISPR-associated sequence (Cas) gene Cas9 as one of the modern genome editing technologies. Due to inconsistent definitions, the existing CRISPR detection programs seem to have missed some weak CRISPR signals.* Results.* This study manually curates all the currently annotated CRISPR elements in the prokaryotic genomes and proposes 95 updates to the annotations. A new definition is proposed to cover all the CRISPRs. The comprehensive comparison of CRISPR numbers on the taxonomic levels of both domains and genus shows high variations for closely related species even in the same genus. The detailed investigation of how CRISPRs are evolutionarily manipulated in the 8 completely sequenced species in the genus* Thermoanaerobacter* demonstrates that transposons act as a frequent tool for splitting long CRISPRs into shorter ones along a long evolutionary history.

## 1. Introduction

A CRISPR is an array of repeat copies (DR, direct repeat) connected by fixed-length linker sequences [1]. The linker sequences are called spacers and are usually acquired from the genetic elements invading the host microbial cells [2]. A CRISPR may be activated by its neighboring CRISPR-associated (Cas) genes, and the spacers will be processed into RNA molecular. The RNA form of spacers will repress the activities of foreign elements with reverse-complementary regions that reinvade the host cells [1–3]. Although CRISPRs are only detected in microbial genomes in the nature, it has been engineered as one of the major genomic editing technologies for both animal and plant genomes [4, 5]. So it is essential to study CRISPR's evolutionary dynamic patterns.

Only a few computational tools were released to automatically detect CRISPRs from a given genome, but they have different default parameter settings for a CRISPR. PILER-CR [6] screens for a repeat array using a local genomic self-alignment and has *O*(*L* ^3^) for the complexities of both time and memory space, where *L* is the genome length. PILER-CR requires the DR length to be between 20 and 40 bps. CRT [7] starts with the scanning for local repetitive *k*-mers, which is a nucleotide sequence with length *k*. Due to its nature of local scanning, CRT runs for linear time and within linear memory space. Its default setting for DR lengths is between 21 and 37 bps. The latest tool CRISPRFinder [8] uses an existing tool Vmatch to find the DR array in a given genome and will discard the tandem repeats as false positives. CRISPRFinder has a slightly longer assumption for DRs between 20 and 47 bps. A comprehensive database DbCRISPR was also published to provide the CRISPR annotations for 2,762 microbial genomes [9].

Due to the different default settings of existing tools for a CRISPR structure, we hypothesize that a comprehensive manual curation may refine the current CRISPR annotations and facilitate the discovery of CRISPR evolutionary mechanisms. This study proposed 95 updated CRISPR annotations, the majorities (59/95~62.11%) of which are transposon-broken CRISPRs. A new CRISPR definition is proposed and all the curated data are available as easy-to-use FASTA/GFF3 formats. The CRISPR variations within all the prokaryotic genus are summarized based on the curated annotations, and the dynamic patterns of CRISPRs in the genus* Thermoanaerobacter* are investigated in detail.

## 2. Material and Methods

### 2.1. Initial CRISPR Annotations

The complete annotation of CRISPRs in microbial genomes was downloaded from the latest version of the database DbCRISPR [9], which was updated on April 14, 2014. 4,065 CRISPRs are annotated in the 2,762 genomes of bacteria and archaea. The questionable structures in DbCRISPR are omitted. If a genome harbors CRISPRs, it has 3.11 CRISPRs on average.

SpacerDB consists of all the annotated CRISPR spacer sequences in the database DbCRISPR and was downloaded from the website of DbCRISPR [9] on April 14, 2014. CRISPR spacers are not random nucleotide sequences and are supposed to originate from the foreign invasive elements like phages [2]. So a DR flanking sequence matching a known spacer may also be a spacer.

### 2.2. Analysis Techniques and Tools

2,762 genomes of bacteria and archaea are identified CRISPR by running CRT, CRISPRFinder, and PILER-CR, respectively. However, lots of CRISPR results are not common on these three software programs. Thus, the CRISPRs in the DbCRISPR are considered the gold standard. The ratios, which are produced by the number of the spacers in CRT, CRISPRFinder, or PILER-CR results to the number of the spacers in the DbCRISPR, are statistically analyzed. If the ratio is greater than 1 or less than 1, the corresponding CRISPRs are manually analyzing, checking, modifying, and correcting the above CRISPR results based on the database DbCRISPR. A comprehensive manual curation was conducted to screen for candidate DRs in the CRISPR flanking regions. For an annotated CRISPR, the homologous copies of DRs were screened by the local copy of NCBI BLAST version 2.2.25 [10]. NCBI BLAST is also used to screen the homologous matches of a given spacer sequence.

A CRISPR is usually activated by the closest CRISPR-associated (Cas) genes [11], and multiple CRISPRs may share the same group of Cas genes, if there is only one such group neighboring to these CRISPRs.

## 3. Results and Discussion

In summary, this study conducts a comprehensive curation of the current CRISPR annotation and proposes three types of revisions based on the observations that some annotated CRISPRs (1) have undetected DRs in the flanking regions, (2) are broken into two CRISPRs due to the nonstandard DRs or transposons in between, or (3) are annotated as two CRISPRs at the beginning of circular chromosomes. The following sections elaborate in detail the three types of annotation errors and demonstrate some interesting observations.

### 3.1. Detection of New DRs and Spacers

A CRISPR is a few copies of a short repeat (DR, direct repeat) gapped by unique linking sequences (spacer) [1], as shown in Figure S1 in Supplementary Material available online at http://dx.doi.org/10.1155/2016/7237053. But different computational annotation programs do not agree on the default values for the lengths of DRs and spacers. The CRISPR annotation database DbCRISPR restricts a DR within 21 to 47 bps in length and a spacer within 25 to 60 bps in length [9]. The same group of authors released a CRISPR detection program, CRISPRFinder [8], which has a different requirement for the DR length (within 23 to 55 bps). And CRISPRFinder also requires the length of a spacer to be within 0.6 to 2.5 times of the DR length. Two other existing programs CRT [7] and PILER-CR [6] require the DR lengths to be within 19 to 38 bps and 16 to 64 bps, respectively.

Lots of CRISPR results are not common on these three software programs CRISPRFinder, CRT, and PILER-CR. Based on the database DbCRISPR, we made novel discoveries by manually analyzing, modifying, and correcting the above CRISPR results and investigated the lengths of CRISPR DRs and spacers. After the corrections of CRISPR annotations in the following sections, we will give a revised CRISPR definition.

**Figure 1:**
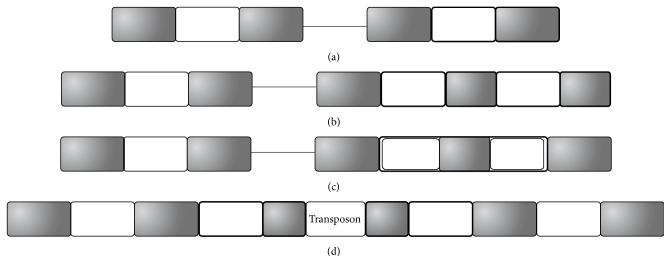
More DRs remain to be detected in the database DbCRISPR. A CRISPR annotation may miss (a) one complete DR or (b) two imperfect DRs in the flanking regions. (c) Some CRISPR spacers may harbor truncated DRs, which are partially identical to the hosting CRISPR's DRs. And (d) many CRISPRs have DRs broken by transposon insertions. DRs and spacers are represented by gray and white boxes, respectively. The new regions added to the DbCRISPR annotations are boxed in bold.

A few DRs were not detected in the flanking regions of CRISPRs, as demonstrated in Figures 1(a) and 1(b). Six CRISPRs may have one missing DR in the flanking region, as in Figure 1(a). For example, by screening for more DRs in the CRISPR flanking regions, we propose 10 spacers for the CRISPR NC_010125_2181482_2182111 in* Gluconacetobacter diazotrophicus *PAl 5, as in Figure S2. But DbCRISPR only detected 9 spacers for this CRISPR. The new DR is also confirmed using the tool CRISPRFinder [8]. Four other CRISPRs (NC_010125_62935_64899, NC_010125_2253748_2255112, NC_011365_388303_388536, and NC_011365_460172_461964) in the same bacterial strain* Gluconacetobacter diazotrophicus *PAl 5 missed one complete DR in their flanking regions too, as in Figure S2.

Two DRs were added to each of 4 CRISPRs, as in Figure 1(b). These DRs were missed by the database DbCRISPR mainly due to the fact that one of the two DRs is only partially identical to the other DRs, as in Figure S3. One of the example CRISPRs is NC_014152_2078344_2080300 in the bacterial genome* Thermincola* sp. JR, with 26 spacers. We propose two more DRs for this CRISPR, although one of the two new DRs is identical to the other DRs in half of its region. The mismatched region may be introduced by the gene conversion [12] or homologous recombination [13] mechanism. Another piece of supporting evidence for the two new spacers comes from their BLAST matches to two known spacers in the other genomes in the SpacerDB with 91.3% and 94.4% in matching identity percentages, respectively. A spacer is supposed to originate from the foreign invasive elements. Since it is low in probability to have such almost identical sequences just by the random single nucleotide mutations, the two new candidate spacers are suggested to be real spacers originated from the same foreign invasive elements as the two homologous copies in the other genomes. Three more CRISPRs, that is, NC_015865_1907425_1908328 in* Thermococcus *sp. 4557, NC_015738_2085666_2087297 in* Eggerthella *sp. YY7918, and NC_014209_791663_793738 in* Thermoanaerobacter mathranii *subsp*. mathranii *str. A3, are expanded with two more DRs for the same reason, as in Figure S3.

**Figure 2:**
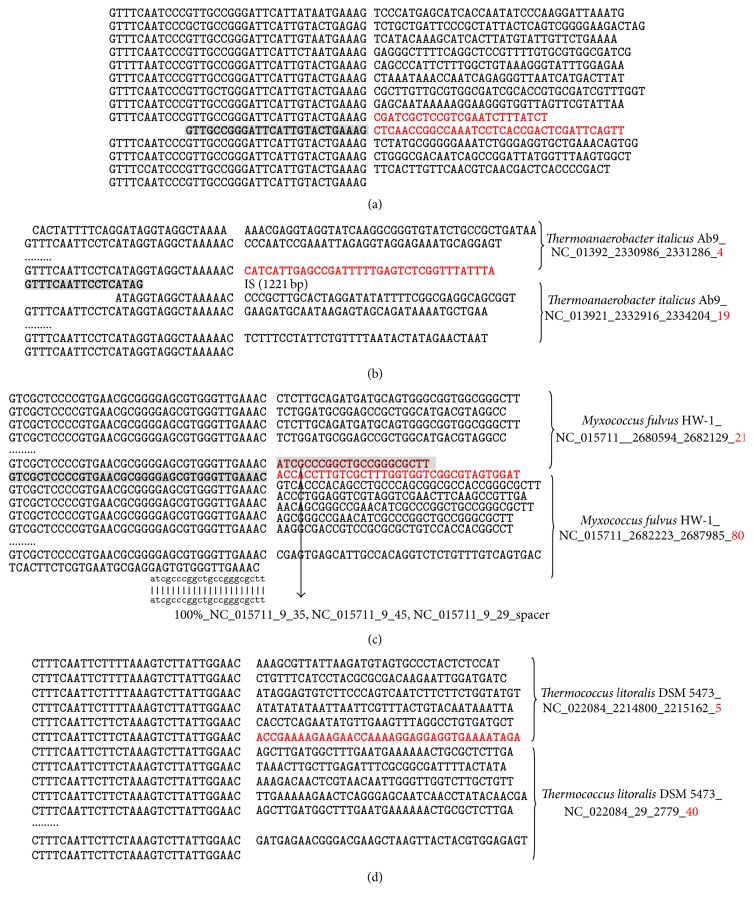
Detection of new DRs and spacers. (a) A CRISPR in* Oscillatoria acuminata* PCC 6304 has a long spacer with a truncated DR inside. The long spacer is in red and the truncated DR is in shade. DRs and spacers are represented in the left and right columns, respectively. (b) A CRISPR with DRs inserted by transposons. The newly annotated spacer regions are in red, and the new DRs are in shade. The last number in the CRISPR name is the DR copy number. (c) A CRISPR with undetected DRs inside. The two neighboring CRISPRs have almost identical DRs and one undetected DR in between. The undetected DR may be a full copy. The added region is highlighted in bold. The regions of new DRs matching the existing DRs are in shade. The regions of new spacers matching the spacers in other genomes are in shade. The last number in the CRISPR name is the DR copy number. (d) A CRISPR is broken into two at the beginning of a circular chromosome of* Thermococcus litoralis* DSM 5473. One more spacer is proposed to combine the two CRISPRs into one longer CRISPR. The added region (spacer) is highlighted in red.

Each of 3 CRISPRs has an extraordinarily long spacer with a truncated DR inside, as demonstrated in Figure 1(c). The representative example is the CRISPR NC_019693_6234891_6235861 with 12 spacers in the cyanobacterial strain* Oscillatoria acuminata* PCC 6304.  Figure 2(a) illustrates that this CRISPR's ninth spacer harbors a partial DR copy with 70% length of the other DRs. And the two flanking sequences in this long spacer have reasonable lengths as spacers. So we propose that this CRISPR has 13 spacers, as in Figure 2(a). Similar cases are detected in two other CRISPRs, that is, NC_008639_1625359_1633049 in* Chlorobium phaeobacteroides* DSM 266 and NC_007777_3904715_3905896 in* Frankia* sp. CcI3, as in Figure S4.

Quite a number of CRISPRs acquired transposon insertions and were broken into two CRISPRs in the DbCRISPR annotations, as in Figure 2(b). All the 59 cases are demonstrated in Figure S5. Our curation shows that there are 59 CRISPRs with flanking DRs inserted by transposons, for example, insertion sequence (IS) elements [14, 15] or miniature inverted-repeat transposable elements (MITEs) [16]. Figure 2(b) illustrates that the 1,221 bp IS element is inserted into the DR sequence of the CRISPR in the genome* Thermoanaerobacter italicus* Ab9. The 4 bp tandem duplication ATAG in the DR sequence also supports that this IS copy was recently translocated here. A 180 bp MITE element is also observed to be within the DR sequence of a CRISPR in* Microcystis aeruginosa* NIES-843, and the 5 bp tandem duplication CTATT flanking the MITE should be produced during its recent translocation, as in Figure S5. Summary of all the 59 transposon insertions in CRISPRs may be found in Figure S5.

Some DRs were not detected in the database DbCRISPR, so that a long CRISPR may be annotated as two neighboring ones with almost identical DRs. 4 CRISPRs have a full DR copy that were not detected in the database DbCRISPR. The representative example is found in the Deltaproteobacteria* Myxococcus fulvus* HW-1. DbCRISPR annotates two neighboring CRISPRs NC_015711_2680594_2682129 and NC_015711_2682223_2687985, with 21 and 80 spacers, respectively. These two CRISPRs have the same DR sequence (GTCGCTCCCCGTGAACGCGGGGAGCGTGGGTTGAAAC) and a 94 bp gap in between, as demonstrated in Figure 2(c). But there is an identical DR copy in the 94 bp gap, which is not detected in the database DbCRISPR due to an unknown reason. The sequence between this DR copy and the annotated CRISPR NC_015711_2680594_2682129 identically matches three spacers in the same genome. A CRISPR spacer is supposed to be acquired from foreign invasive elements [1], and the data suggests that the microbial defense system CRISPR has generated four spacers to respond to this foreign element. The shared Cas genes further support that NC_015711_2680594_2682129 and NC_015711_2682223_2687985 may be joined by the 94 bp gap as one longer CRISPR. Three other similar cases were detected in the bacteria* Caldicellulosiruptor obsidiansis *OB47,* Thermosipho africanus* TCF52B, and* Herpetosiphon aurantiacus *ATCC 23779, as shown in Figure S6. These DRs were missed mainly due to their short lengths slightly below the threshold of spacer/DR ∈[0.6,2.5]. 11 other CRISPRs were broken mainly due to an internal partial DR copy that was not detected by the database DbCRISPR, as shown in Figure S6.

Unlike the eukaryotic counterparts, most of the microbial chromosomes are in the circular shape [17], but the database DbCRISPR regards a CRISPR spanning the beginning point of a circular chromosome as two. We manually checked the 4,065 CRISPRs annotated in the database DbCRISPR and detected 8 such cases.  Figure 2(d) shows two CRISPRs NC_022084_2214800_2215162 and NC_022084_29_2779 in the archaea* Thermococcus litoralis* DSM 5473, with 5 and 40 spacers, respectively. The identical DRs and the shared Cas genes suggest that these two closely located CRISPRs may be joined into one by the 38 bp sequence between them. This updated information is important, since the missing spacer may be a key anti-invasion factor. 7 other cases were detected in the database DbCRISPR, as demonstrated in Figure S7.

### 3.2. An Updated CRISPR Definition

Based on the curated annotations of all the CRISPRs in the prokaryotic genomes, this study proposes an updated definition of a CRISPR, as demonstrated in Figure 1 and summarized in Table 1. The minimum number of DRs in a CRISPR may be as low as 2. And a DR may have a length between 14 and 55 bps. A spacer is 9–95 bps in length. The length ratio between a spacer and a DR is proposed to be between 0.3 and 2.5.

The three previous CRISPR annotation programs do not have a consensus agreement on the range of a DR length. In the default settings, CRT, CRISPRFinder, and PILER-CR assume that a DR is at least 19, 23, and 16 bps, respectively. But the Cyanobacteria* Microcystis aeruginosa *NIES 843 and the Firmicutes* Thermacetogenium phaeum* DSM 12270 have a CRISPR with the minimum DR lengths of 14 and 15, respectively. The program CRT requires a DR to be at least 19 bps in length, which will miss CRISPRs with a short 17 bp DR in the 7 Firmicutes and an Actinobacteria genomes. The maximum DR length observed in the curated CRISPR annotations is 55 bps. So the program PILER-CR's default value for this feature 64 bps is not strictly supported by the observations. CRT requires the maximum CRISPR DR to be at most 38 bps, which will not recognize CRISPRs in the 30 bacterial genomes. CRISPRFinder has the same setting with caCRISPR for the maximum DR length of 55 bps.

This study proposes the range of a spacer length in two measurements, that is, 9–95 bps and 0.3–2.5 DRs. The program CRT assumes a spacer to be at least 19 bps in length, which will miss CRISPRs in the four Archaea Crenarchaeota genomes and 21 bacterial genomes (14/21~66.67% are Proteobacteria). CRISPRs in the 191 and 49 prokaryotic genomes will not be recognized by the programs CRT and PILER-CR due to their assumptions of the maximum spacer lengths of 48 and 64 bps, respectively. CRISPRFinder has the same requirement as caCRISPR for the maximum spacer length as 2.5 DRs, but its assumption of a minimum spacer length 0.6 DR will miss a CRISPR with the minimum spacer/DR ratio 0.594 and CRISPRs in the 15 bacterial genomes. So the data suggests that the spacer length in two measurements will provide higher specificity and cover all the known CRISPRs.

For the convenience of further analysis, the curated CRISPR annotations are released in the formats of both FASTA and GFF3 in the Supplementary Materials. Other file formats may be provided upon request.

### 3.3. Taxonomical Distributions of CRISPRs in Prokaryotic Genomes

**Figure 3:**
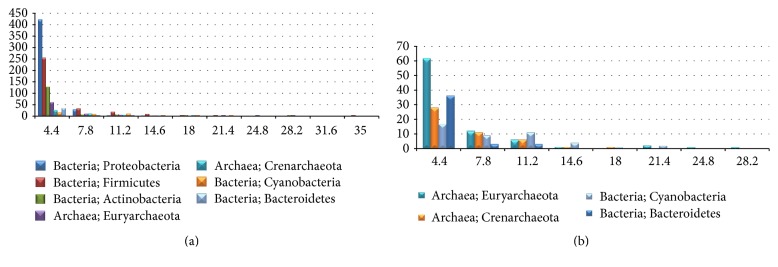
Histograms of the genome numbers (in vertical axis) versus the CRISPR numbers per genome (in horizontal axis). The summaries are conducted for the taxonomic domains of (a) at least 30 genomes and (b) 30–100 genomes, each of which has at least one CRISPR.

Among the 4,052 annotated CRISPRs in the 1,302 genomes, the majority comes from the 7 domains (1,149/1,302~88.25%). The seven domains of genomes harbor 3,458/4,052~85.34% of the known CRISPRs, and the 460 (460/1,302~35.33%) Proteobacteria alone have 25.42% (~1,030/4,052) of the annotated CRISPRs. Firmicutes is the second largest domain of the annotated CRISPRs, and 1,030 CRISPRs come from the 323 Firmicutes bacterial genomes. Another 148 CRISPRs are detected in the 411 actinobacterial genomes. And Figure 3(a) shows that Firmicutes tends to have more CRISPRs in one genome, since Firmicutes has more genomes with at least 4.4 CRISPRs (the upper limit of the first bin) than any other domains.

After excluding the top three domains of genomes with CRISPRs, the other four largest domains of CRISPRs are the two archaea domains Euryarchaeota and Crenarchaeota and the other two bacteria domains Cyanobacteria and Bacteriodetes, as shown in Figure 3(b). All the three domains show the trend that the number of genomes decreases with the increased CRISPR number per genome, except the domain Cyanobacteria. The number of Cyanobacteria genomes (11) in the third bin is larger than that (9) in the second bin, as shown in Figure 3(b), suggesting that cyanobacterial genomes tend to harbor a large number of CRISPRs. Actually Cyanobacteria is the domain with the maximum average CRISPR number per genome (6.71 for the 44 genomes), if the bacterial domain Fibrobacteres is omitted. There is only one species* Fibrobacter succinogenes *subsp.* succinogenes* S85 in this domain, and 29 CRISPRs are annotated in this genome.

### 3.4. Dynamic Patterns of CRISPRs in the Prokaryotic Genomes

**Figure 4:**
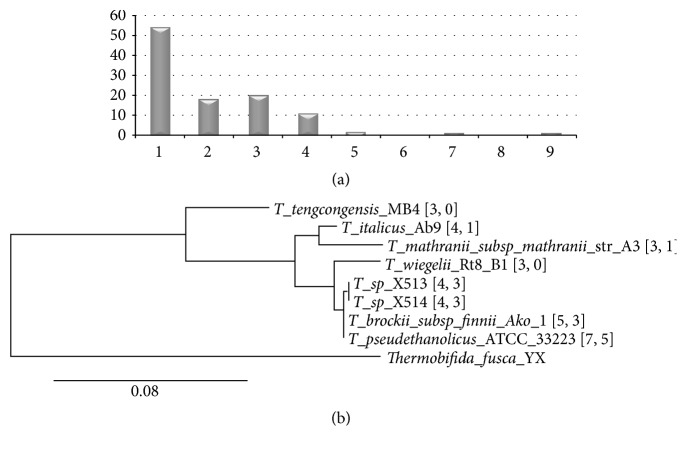
CRISPR distribution dynamics in the prokaryotic genomes. (a) Histogram of the standard deviation (StdEv) values of the 107 genera, each of which has at least three genomes in the CRISPR annotations. (b) The 16S rRNA phylogenetic tree of the 8 completely sequenced* Thermoanaerobacter *genomes, rooted at a closely related species* Thermobifida fusca YX*. The two numbers in the brackets are the numbers of annotated CRISPRs and CRISPRs with transposon insertions, respectively.

CRISPRs show significant variations in its distributions between closely related prokaryotic genomes. We calculate the standard deviation (StdEv) of CRISPR number per genome in the genus with at least three genomes in the annotations. 54 of the 107 genera have a StdEv smaller than 1.0, as shown in Figure 4(a), and only 8 genera demonstrate StdEv = 0, suggesting that these closely related genomes have the same numbers of CRISPRs. 38 genera also show variable CRISPR numbers, with StdEv ∈(1.0,3.0]. The archaea genus* Methanocaldococcus *evens reaches StdEv = 8.26 for the CRISPR numbers in its six genomes, ranging from 1 to 22.

We further investigate the CRISPR number variations among different species of the same genus* Thermoanaerobacter*, for its high appearing rate in the CRISPR annotation corrections.  Figure 4(b) illustrates how actively CRISPRs in the 8 completely sequenced species of* Thermoanaerobacter* are manipulated by the transposon insertions. Six of the eight species carry CRISPRs inserted by Insertion Sequences (IS) or Miniature Inverted-Repeat Transposable Elements (MITE), as shown in Figure S4. Four CRISPRs in the two genomes* T.* sp. X513 and X514 originate from the same long CRISPR inserted by three copies of the transposon IS110, according to the evidences of almost identical DR sequences and spacers with similar lengths. The same insertion flanking sequences and close phylogenetic distance suggest that the three insertions happen in the common ancestor of these two species. After the divergence of the two genomes, the fourth CRISPR in* T. *sp. X514 continues to expand with 14 more spacers. IS110 also plays an active role in splitting a long CRISPR into shorter ones in the two other* Thermoanaerobacter* genomes, that is,* T. brockii *subsp*. finnii* Ako-1 and* T. pseudethanolicus* ATCC 33223. A long CRISPR in* T. brockii *subsp*. finnii* Ako-1 is broken into four by two insertions of IS110 and one IS1634 insertion. The long CRISPR in* T. pseudethanolicus* ATCC 33223 shares the same DR sequence and spacers with similar lengths but undergoes more diversified manipulations, as shown in Figure S4. Besides the three single-copy IS110 insertions, an IS110 dimer and a Miniature Inverted-Repeat Transposable Element (MITE) are also detected in the split of this long CRISPR into shorter ones. These data demonstrate that CRISPRs in the* Thermoanaerobacter* genomes are under active evolutionary manipulation and expansion.

## Supplementary Material

The detailed information of CRISPRs and all the newly found DRs and spacers are included in the Supplementary Material. The definition structure of a CRISPR is given in the Supplementary Figure S1. The CRISPR annotations corrected by different rules were illustrated in the Supplementary Figures S2-S7. Supplementary Figures S2 and S3 illustrate the 6 cases with one new DR, and 4 cases with two new DRs, respectively. Supplementary Figure S4 describes 3 cases with partial DRs covered by long spacers. Supplementary Figures S5 and S6 illustrate 59 cases with DRs broken by transposons, 15 more cases broken by undetected internal DRs. Supplementary Figure S7 describes 8 CRISPRs broken due to that a circular microbial chromosome is recorded as a linear DNA sequence.

## Figures and Tables

**Table 1:** Summary of CRISPR definitions in different studies.

# program	minDR #	minDR	maxDR	minSpacer	maxSpacer
CRT	3	19 bp	38 bp	19 bp	48 bp
CRISPRFinder	3	23 bp	55 bp	0.6 DR	2.5 DR
PILER-CR	3	16 bp	64 bp	8 bp	64 bp
*caCRISPR*	*3*	*14 bp*	*55 bp*	*9 bp (0.3 DR)*	*95 bp (2.5 DR)*

Column “minDR #” gives the minimum number of DRs required to define a CRISPR. Columns “minDR” and “maxDR” are the minimum and maximum DR lengths. The other two columns “minSpacer” and “maxSpacer” give the minimum and maximum spacer lengths for a CRISPR. The proposed definition for a CRISPR in this study is denoted as “caCRISPR,” and the other three computer programs compared in this study are CRT (CRISPR Recognition Tool) [7], CRISPRFinder [8], and PILER-CR [6].
