# Supplementary material for: A Comprehensive Curation Shows the Dynamic Evolutionary Patterns of Prokaryotic CRISPRs
Source: Biomed Res Int. 2016 Apr 18;2016:7237053. doi: 10.1155/2016/7237053 (PMC4852346; doi:10.1155/2016/7237053)
Supplement: Supplementary file 1 — The detailed information of CRISPRs and all the newly found DRs and spacers are included in the Supplementary Material. The definition structure of a CRISPR is given in the Supplementary Figure S1. The CRISPR annotations corrected by different rules were illustrated in the Supplementary Figures S2-S7. Supplementary Figures S2 and S3 illustrate the 6 cases with one new DR, and 4 cases with two new DRs, respectively. Supplementary Figure S4 describes 3 cases with partial DRs covered by long spacers. Supplementary Figures S5 and S6 illustrate 59 cases with DRs broken by transposons, 15 more cases broken by undetected internal DRs. Supplementary Figure S7 describes 8 CRISPRs broken due to that a circular microbial chromosome is recorded as a linear DNA sequence. [file 7237053.f1.pdf]

A comprehensive curation shows the dynamic evolutionary patterns of prokaryotic CRISPRs

Guoqin Mai[1,*], Ruiquan Ge[1,2,*], Guoquan Sun[1], Qinghan Meng[1,2], Fengfeng Zhou[3,1,#]

[1] Shenzhen Institutes of Advanced Technology, and Key Lab for Health Informatics, Chinese Academy of Sciences,

[2] Shenzhen College of Advanced Technology, University of Chinese Academy of Sciences, Shenzhen, Guangdong, P.R. China, 518055.

[3] College of Computer Science and Technology, Jilin University, Changchun, Jilin, P.R. China, 130012.

* These authors contribute equally to this work.

[#]To whom correspondence should be addressed. Corresponding author: Fengfeng Zhou, e-mail: FengfengZhou@gmail.com, or ffzhou@jlu.edu.cn. Web site: http://www.healthinformaticslab.org/ffzhou/.

Figure S1. Structure of a CRISPR.

Structure of a CRISPR. DR is the direct repeat flanking the spacer sequence. The grey box represents the DR sequence, and DRs of the same CRISPR are homologous to each other.The patterned boxes represent the spacer sequence.

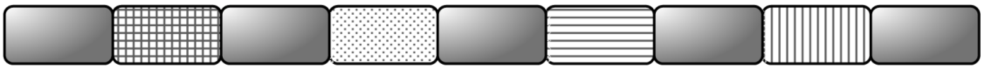

Figure S2. 6 cases with one new DR

(a) CRISPRdb only detected 9 spacers for a CRISPR (NC_010125_ 2181482_2182111) in the *Gluconacetobacter diazotrophicus* PAl 5, but we detected 10 spacers for this CRISPR (NC_010125_2181482_2182177). **(b)** CRISPRdb only detected 28 spacers for a CRISPR (NC_010125_ 62935_64832) in the *Gluconacetobacter diazotrophicus* PAl 5, but we detected 29 spacers for this CRISPR (NC_010125_62935_64899). **(c)** CRISPRdb only detected 21 spacers for a CRISPR (NC_010125_ 2253747_2255055) in the *Gluconacetobacter diazotrophicus* PAl 5, but we detected 22 spacers for this CRISPR (NC_010125_2253747_2255112). **(d)** CRISPRdb only detected 28 spacers for a CRISPR (NC_011365_460172_461907) in the *Gluconacetobacter diazotrophicus* PAl 5, but we detected 29 spacers for this CRISPR (NC_011365_460172_461964_29). **(e)** CRISPRdb only detected 3 spacers for a CRISPR(NC_011365_388303_388536) in the *Gluconacetobacter diazotrophicus* PAl 5, but we detected 4 spacers for this CRISPR (NC_011365_ 388303_388602). **(f)** CRISPRdb only detected 96 spacers for a CRISPR (NC_008553_670839_677982) in the *Gluconacetobacter diazotrophicus* PAl 5, but we detected 97 spacers for this CRISPR (NC_008553_670839_677982). The new spacers are marked by yellow background, and the new DRs are highlighted in red.

> *Gluconacetobacter diazotrophicus* PAl 5_NC_010125_2181482_2182177_10_our results

```
AGCCTACCATCGGCAAATCGGTAGGGAAACCACGGC    CTGGCCGGTTAATTGCGTGACGGCGGGTTC
AGCCTACCATCGGCAAATCGGTAGGGAAACCACGGC    ATCGCATGACCTTTGGTTTCGACCGGGTAT
AGCCTACCATCGGCAAATCGGTAGGGAAACCACGGC    TCGAAGACCGCGCTGGACGACATGGGGAAG
AGCCTACCATCGGCAAATCGGTAGGGAAACCACGGC    CGATTCCGATACCTTGCGCGTGCGCACTGG
AGCCTACCATCGGCAAATCGGTAGGGAAACCACGGC    GCAAGATAACGGCCTTCGGCCACACGAAGA
AGCCTACCATCGGCAAATCGGTAGGGAAACCACGGC    GCAGATTTCGATACCGGCAACGACGGTCTT
AGCCTACCATCGGCAAATCGGTAGGGAAACCACGGC    GAGCGGAGGCAGCTTGTGGCCCAATATGGC
AGCCTACCATCGGCAAATCGGTAGGGAAACCACGGC    GATCGCGCATGATGGCCGGATCAACGCTCA
AGCCTACCATCGGCAAATCGGTAGGGAAACCACGGC    ATCGAGCGCCGGCCGGACGTCGTTGTCGCC
AGCCTACCATCGGCAAATCGGTAGGGAAACCACGGC    CATGGTGTCGAGCTTGCTCGGCGCGTTCTC
AGCCTACCATCGGCAAATCGGTAGGGAAACCACGGC
```

> *Gluconacetobacter diazotrophicus* PAl 5_NC_010125_ 2181482_2182111_9_gold standard

```
AGCCTACCATCGGCAAATCGGTAGGGAAACCACGGC    CTGGCCGGTTAATTGCGTGACGGCGGGTTC
AGCCTACCATCGGCAAATCGGTAGGGAAACCACGGC    ATCGCATGACCTTTGGTTTCGACCGGGTAT
AGCCTACCATCGGCAAATCGGTAGGGAAACCACGGC    TCGAAGACCGCGCTGGACGACATGGGGAAG
AGCCTACCATCGGCAAATCGGTAGGGAAACCACGGC    CGATTCCGATACCTTGCGCGTGCGCACTGG
AGCCTACCATCGGCAAATCGGTAGGGAAACCACGGC    GCAAGATAACGGCCTTCGGCCACACGAAGA
AGCCTACCATCGGCAAATCGGTAGGGAAACCACGGC    GCAGATTTCGATACCGGCAACGACGGTCTT
AGCCTACCATCGGCAAATCGGTAGGGAAACCACGGC    GAGCGGAGGCAGCTTGTGGCCCAATATGGC
AGCCTACCATCGGCAAATCGGTAGGGAAACCACGGC    GATCGCGCATGATGGCCGGATCAACGCTCA
AGCCTACCATCGGCAAATCGGTAGGGAAACCACGGC    ATCGAGCGCCGGCCGGACGTCGTTGTCGCC
AGCCTACCATCGGCAAATCGGTAGGGAAACCACGGC
```

> *Gluconacetobacter diazotrophicus* PAl5_NC_010125_62935_64899_29_ our results

GTTTTAATCCCCGCTTCCGCGTGGGGAGCGAC	GACAACCTTGCGACTCCTGTCCGACGCGGTCGGC
GTTTCAATCCACGCTCCCGCACGGGGAGCGAC	GGGTGCGATCCTCCGCATGGTGCAGGAGGACATC
GTTTCAATCCACGCTCCCGCACAGGGAGCGAC	CGCCTCGTCGATGATGACGGGCGAATTCATCACGCC
GTTTCAATCCACGCTCCCGCACAGGGAGCGAC	GATCACGCGCACGCACCGCAGCCACTCGTCGTAGG
GTTTCAATCCACGCTCCCGCACAGGGAGCGAC	CAACTGTCCGATATCTCGGCCAATCTCTCCAACG
GTTTCAATCCACGCTCCCGCACAGGGAGCGAC	CGTTTCTCGATACGCGGTCCAATCCCCTCCTTCTT
GTTTCAATCCACGCTCCCGCACAGGGAGCGAC	CAACGTCTCCGACCCTTGACGTGGTCTCACGCCT
GTTTCAATCCACGCTCCCGCACAGGGAGCGAC	GAGCCGGGGACGAGCGTGTAATCCTGCTGCGCCG
………	
GTTTCAATCCACGCTCCCGCACAGGGAGCGAC	CTTCTGTTCGGCATCGCCGGCGCGTCTGGTTCCG
GTTTCAATCCACGCTCCCGCACAGGGAGCGAC	AAGATCCATGTCCTTCCAAGCGCATTCCCTAAAT
GTTTCAATCCACGCTCCCGCACAGGGAGCGAC	GGGAAACGAACCGTGATGTCGTGGCGGAAAGAAAG
GTTTCAATCCACGCTCCCGCACAGGGAGCGAC	

> *Gluconacetobacter diazotrophicus* PAl5_NC_010125_ 62935_64832_28_gold standard

GTTTTAATCCCCGCTTCCGCGTGGGGAGCGAC	GACAACCTTGCGACTCCTGTCCGACGCGGTCGGC
GTTTCAATCCACGCTCCCGCACGGGGAGCGAC	GGGTGCGATCCTCCGCATGGTGCAGGAGGACATC
GTTTCAATCCACGCTCCCGCACAGGGAGCGAC	CGCCTCGTCGATGATGACGGGCGAATTCATCACGCC
GTTTCAATCCACGCTCCCGCACAGGGAGCGAC	GATCACGCGCACGCACCGCAGCCACTCGTCGTAGG
GTTTCAATCCACGCTCCCGCACAGGGAGCGAC	CAACTGTCCGATATCTCGGCCAATCTCTCCAACG
GTTTCAATCCACGCTCCCGCACAGGGAGCGAC	CGTTTCTCGATACGCGGTCCAATCCCCTCCTTCTT
GTTTCAATCCACGCTCCCGCACAGGGAGCGAC	CAACGTCTCCGACCCTTGACGTGGTCTCACGCCT
GTTTCAATCCACGCTCCCGCACAGGGAGCGAC	GAGCCGGGGACGAGCGTGTAATCCTGCTGCGCCG
………	
GTTTCAATCCACGCTCCCGCACAGGGAGCGAC	CTTCTGTTCGGCATCGCCGGCGCGTCTGGTTCCG
GTTTCAATCCACGCTCCCGCACAGGGAGCGAC	AAGATCCATGTCCTTCCAAGCGCATTCCCTAAAT
GTTTCAATCCACGCTCCCGCACAGGGAGCGAC	

> *Gluconacetobacter diazotrophicus* PAl5_NC_010125_2253747_2255112_22_our results

AGATTCATCCCTGCATATGCGAGGAACAC	AAGGCGGCCTCGTCCAGGATCACCAGACCCTG
CGGTTCATCCCCGCACGTGCGGGAACACG	TCCCCCTGCTCGGCAACCCCAAACTCAAGAT
CGGTTCATCCCCGCACGTGCGGGGAACAC	GCCGCGACGGTGAAGGGGAGGTTGTTCGACGC
CGGTTCATCCCCGCACGTGCGGGGAACAC	GAGTCACCGGATTCCCATCTCATCCTAATATC
CGGTTCATCCCCGCACGTGCGGGGAACAC	CTTTTCTCAGCCGACACTCTCCATGCAAGAAC
CGGTTCATCCCCGCACGTGCGGGGAACAC	ACGCGGGTCTATGAGATTCCGATCATGCAGGA
CGGTTCATCCCCGCACGTGCGGGGAACAC	CATTGACCCGGCACCCTCCCCATGTGACGTTT
CGGTTCATCCCCGCACGTGCGGGGAACAC	TGGCTCGGGACCATCTTAGCGGACGACATGGC
………	
CGGTTCATCCCCGCACGTGCGGGGAACAC	TTGCGTCAAACGAAGCACAATATCAGGCGCAG
CGGTTCATCCCCGCACGTGCGGGGAACAC	TTGCCTCTAACGAGGCGCAGTATCAGGCACAG
CGGTTCATCCCCGCACGTGCGGGGAACAC	CCGTCGTTTCGATCGTCACGGCGATCATCGGC
CGGTTCATCCCCGCACGTGCGGGGA	

> *Gluconacetobacter diazotrophicus* PAl5_NC_010125_ 2253747_2255055_21_gold standard

AGATTCATCCCTGCATATGCGAGGAACAC	AAGGCGGCCTCGTCCAGGATCACCAGACCCTG
CGGTTCATCCCCGCACGTGCGGGAACACG	TCCCCCTGCTCGGCAACCCCAAACTCAAGAT
CGGTTCATCCCCGCACGTGCGGGGAACAC	GCCGCGACGGTGAAGGGGAGGTTGTTCGACGC
CGGTTCATCCCCGCACGTGCGGGGAACAC	GAGTCACCGGATTCCCATCTCATCCTAATATC
CGGTTCATCCCCGCACGTGCGGGGAACAC	CTTTTCTCAGCCGACACTCTCCATGCAAGAAC
CGGTTCATCCCCGCACGTGCGGGGAACAC	ACGCGGGTCTATGAGATTCCGATCATGCAGGA
CGGTTCATCCCCGCACGTGCGGGGAACAC	CATTGACCCGGCACCCTCCCCATGTGACGTTT
CGGTTCATCCCCGCACGTGCGGGGAACAC	TGGCTCGGGACCATCTTAGCGGACGACATGGC
………	
CGGTTCATCCCCGCACGTGCGGGGAACAC	TTGCGTCAAACGAAGCACAATATCAGGCGCAG
CGGTTCATCCCCGCACGTGCGGGGAACAC	TTGCCTCTAACGAGGCGCAGTATCAGGCACAG
CGGTTCATCCCCGCACGTGCGGGGAACAC	

> *Gluconacetobacter diazotrophicus* PAl5_NC_011365_460172_461964_29_our results

AGATTCATCCCTGCATATGCGAGGA	ACACAAGGCGGCCTCGTCCAGGATCACCAGACCCTG
CGGTTCATCCCCGCACGTGCGGGAA	CACGTCCCCCTGCTCGGCAACCCCAAACTCAAGAT
CGGTTCATCCCCGCACGTGCGGGGA	ACACGCCGCGACGGTGAAGGGGAGGTTGTTCGACGC
CGGTTCATCCCCGCACGTGCGGGGA	ACACCCTGATCCTGCGCCGACGCCGGAACCAGTCCG
CGGTTCATCCCCGCACGTGCGGGGA	ACACGCCGCGACGGTGAAGGGGAGGTTGTTCGACGC
CGGTTCATCCCCGCACGTGCGGGGA	ACACCCTGATCCTGCGCCGACGCCGGAACCAGTCCG
CGGTTCATCCCCGCACGTGCGGGGA	ACACGAGTCACCGGATTCCCATCTCATCCTAATATC
CGGTTCATCCCCGCACGTGCGGGGA	ACACCTTTTCTCAGCCGACACTCTCCATGCAAGAAC
………	
CGGTTCATCCCCGCACGTGCGGGGA	ACACAACGTGGAATGGCACCAGATCGAAACGATGCT
CGGTTCATCCCCGCACGTGCGGGGA	ACACGCGAAGACCTGCGCCTGGAGCGCCTGCTTGTG
CGGTTCATCCCCGCACGTGCGGGGA	ACACCGTGGGGAATATCCTGACACACACATGGGCGT
CGGTTCATCCCCGCACGTGCGGGGA	

> *Gluconacetobacter diazotrophicus* PAl5_NC_011365_460172_461907_28_gold standard

AGATTCATCCCTGCATATGCGAGGA	ACACAAGGCGGCCTCGTCCAGGATCACCAGACCCTG
CGGTTCATCCCCGCACGTGCGGGAA	CACGTCCCCCTGCTCGGCAACCCCAAACTCAAGAT
CGGTTCATCCCCGCACGTGCGGGGA	ACACGCCGCGACGGTGAAGGGGAGGTTGTTCGACGC
CGGTTCATCCCCGCACGTGCGGGGA	ACACCCTGATCCTGCGCCGACGCCGGAACCAGTCCG
CGGTTCATCCCCGCACGTGCGGGGA	ACACGCCGCGACGGTGAAGGGGAGGTTGTTCGACGC
CGGTTCATCCCCGCACGTGCGGGGA	ACACCCTGATCCTGCGCCGACGCCGGAACCAGTCCG
CGGTTCATCCCCGCACGTGCGGGGA	ACACGAGTCACCGGATTCCCATCTCATCCTAATATC
CGGTTCATCCCCGCACGTGCGGGGA	ACACCTTTTCTCAGCCGACACTCTCCATGCAAGAAC
………	
CGGTTCATCCCCGCACGTGCGGGGA	ACACAACGTGGAATGGCACCAGATCGAAACGATGCT
CGGTTCATCCCCGCACGTGCGGGGA	ACACGCGAAGACCTGCGCCTGGAGCGCCTGCTTGTG
CGGTTCATCCCCGCACGTGCGGGGA	

> *Gluconacetobacter diazotrophicus* PAl5_ NC_011365_ 388303_388602 _4 _our results

AGCCTACCATCGGCAAATCGGTAGGGAAACCACGGC	TCAGCCGGCTGGGCATTGCCCTACGAGAGG
AGCCTACCATCGGCAAATCGGTAGGGAAACCACGGC	AGGGGAGCGCGTCACCTTCGACAAGGGCCT
AGCCTACCATCGGCAAATCGGTAGGGAAACCACGGC	CCTCGTTGCTCGCGCGCGGGTGCAGGGCTG
AGCCTACCATCGGCAAATCGGTAGGGAAACCACGGC	TCCGCACGTTCACGCGTACCAGCATCGCGG
AGCCTACCATCGGCAAATCGGTAGGGAAACCACGGC	

> *Gluconacetobacter diazotrophicus* PAl5_ NC_011365_388303_388536_3_gold standard

AGCCTACCATCGGCAAATCGGTAGGGAAACCACGGC	TCAGCCGGCTGGGCATTGCCCTACGAGAGG
AGCCTACCATCGGCAAATCGGTAGGGAAACCACGGC	AGGGGAGCGCGTCACCTTCGACAAGGGCCT
AGCCTACCATCGGCAAATCGGTAGGGAAACCACGGC	CCTCGTTGCTCGCGCGCGGGTGCAGGGCTG
AGCCTACCATCGGCAAATCGGTAGGGAAACCACGGC	

```
GTCGAAGAGCGAGTTCCAGGAAAACAAGGATTGAAAC   TTTCCGGATTAGAATAATAAGGAACTTCCGGTACGGG
GTCGAAGAGCGAGTTCCAGGAAAACAAGGATTGAAAC   GGTATACTAAGCGTACCGTTCGTCTCAACATCGACTA
GTCGAAGAGCGAGTTCCAGGAAAACAAGGATTGAAAC   ACACCATATGCTCCGGGCAGAGGAGCATGTTCACACT
GTCGAAGAGCGAGTTCCAGGAAAACAAGGATTGAAAC   CCAGTCCTCCTCCTCAGGAGGACTGGCTTTTTCAAA
GTCGAAGAGCGAGTTCCAGGAAAACAAGGATTGAAAC   CACAATAAATCCCCCTTTGAAAACGAGTTCAAGC
GTCGAAGAGCGAGTTCCAGGAAAACAAGGATTGAAAC   TATGTAGCTGCATATCTACACCTCCACGGATATAAGAG
GTCGAAGAGCGAGTTCCAGGAAAACAAGGATTGAAAC   TCTTCTGGCTCTGGCACGGCGAGAACTATCTCGCCGT
GTCGAAGAGCGAGTTCCAGGAAAACAAGGATTGAAAC   TCCAAATTACCAAGACAAACATGCTTGTCATGGGTG
………
GTCGAAGAGCGAGTTCCAGGAAAACAAGGATTGAAAC   GCCCATATCTCTTCGTGATGCTCCGCGGTCCTGCTG
GTCGAAGAGCGAGTTCCAGGAAAACAAGGATTGAAAC   AGCAGCATCTTTTTTCACCTCATGATATCATTATGAC
GTCGAAGAGCGAGTTCCAGGAAAACAAGGATTGAAAC   TTTCTATACTGGGGATGTTGTACTTCCTTGCCTTCCTT
GTCGAAGAGCGAGTTCCAGGAAAACAAGGATTGAAAC
```

> *Methanosaeta thermophila* PT_ NC_008553_670839_677982_97 _our results

> *Methanosaeta thermophila* PT _ NC_008553_670839_677982_96 _gold standard

Figure S3. 4 cases with two new DRs

(a) CRISPRdb only detected 26 spacers for a CRISPR (NC_014152_2078344_2080300) in the *Thermincola sp.* JR, but we propose two more DRs for this CRISPR, , although one of the two new DRs is identical to the other DRs in half of its region. These two new spacers comes from their BLAST matches to two known spacers in the other genomes in the SpacerDB with 91.3% and 94.4% in matching identity percentages, respectively. The CRISPRs, *i.e.* NC_015865_1907425_1908328 in *Thermococcus sp.* 4557 **(b)**, NC_015738_2085666_2087297 in *Eggerthella sp.* YY7918 **(c)**, and NC_014209_791663_793738 in *Thermoanaerobacter mathranii subsp. mathranii str.* A3 **(d)**, are also expanded with two more DRs. The new spacers are marked by yellow background, and the new DRs are highlighted in red.

> *Thermincola sp.* JR_NC_014152_2078201_2080300_28_our results

```
 TTTCCGTCCCCTTACGGGGATAAGGAAGATTAAAC   TCTTTGGCAAAGGCAAACATCAGTTGACAGTTGACA
 TTAACAGTTAACAGTTGAGATAAGGAAGATCAAAC   TCTTTGGCAAAGGCAAATATATCATTTTCGGCAACCCAAA
 TTTCCGTCCCCTTACGGGGATAAGGAAGATTAAAC   GCAAACCCGCCGGAACAGGCCGCCGGGGGATGGGACC
GTTTCCGTCCCCTTACGGGGATAAGGAAGATTAAAC   TAAATTCCGAATGGATACTATTTCTTGTCCAGATGCTG
………
GTTTCCGTCCCCTTACGGGGATAAGGAAGATTAAAC   ATGAATTAAGGATGGAATAGTTAATTCCTGCACAGT
GTTTCCGTCCCCTTACGGGGATAAGGAAGATTAAAC
```

146bp

Thermincola sp. JR_NC_014152_
_2078344_2080300_26_gold standard

```
aaggcaaacatcagttgacagtt
||||||||||||| ||| |||||
aaggcaaacatcaattg-cagtt   91.3%_NC_003106_6_11_spacer
```

```
gcaaaggcaaatatatca
||||||||||| ||||||
gcaaaggcaaa-atatca   94.4%_NC_015555_4_102_spacer
```

> *Thermococcus sp.* 4557_NC_015865_1907425_1908328_13_our results

```
GTTGCAATAAGACTCTGGGAGAATTGAAAG        TCGGGAACGCCCAAGTTTATTACGCGGCCGATTACCT
GTTGCAATAAGACTCTGGGAGAATTGAAAG        CGGTTGGCTCATCGGTAGAAACGGGCTCATCTATAATC
………
GTTGCAATAAGACTCTGGGAGAATTGAAAG        CAGGCGCTCAGGGCCGGGGACATACGGGTCGGCCG
GTTGCAACAAGACTCTCTAGGAGAATTGAAA       TGGTGACGATGATGGCCCTGTCCTCCCTGCTCCACTTGG
GTTGCAATAAGACCCCCAGGAGAATCGAAAG
```

Thermococcus sp. 4557_NC_015865_
1907425_1908192_11 _gold standard

136bp

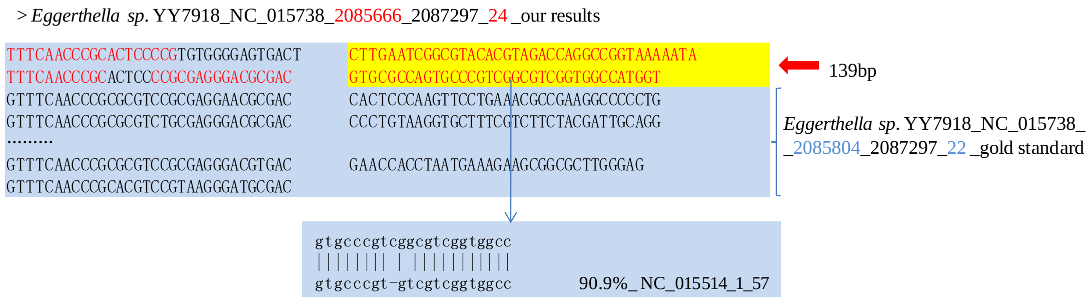

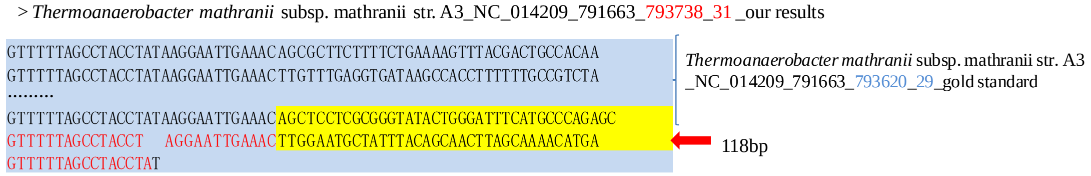

Figure S4. 3 cases of long spacers with a partial DR

(a) A CRISPR in *Oscillatoria acuminata* PCC 6304 has a long spacer with a truncated DR inside. **(b)** A CRISPR in *Chlorobium phaeobacteroides* DSM 266 has a long spacer with a truncated DR inside. **(c)** A CRISPR in *Frankia* sp. CcI3 has a long spacer with a truncated DR inside. The long spacer is in yellow and the truncated DR is in red. DRs and spacers are represented in the left and right columns, respectively.

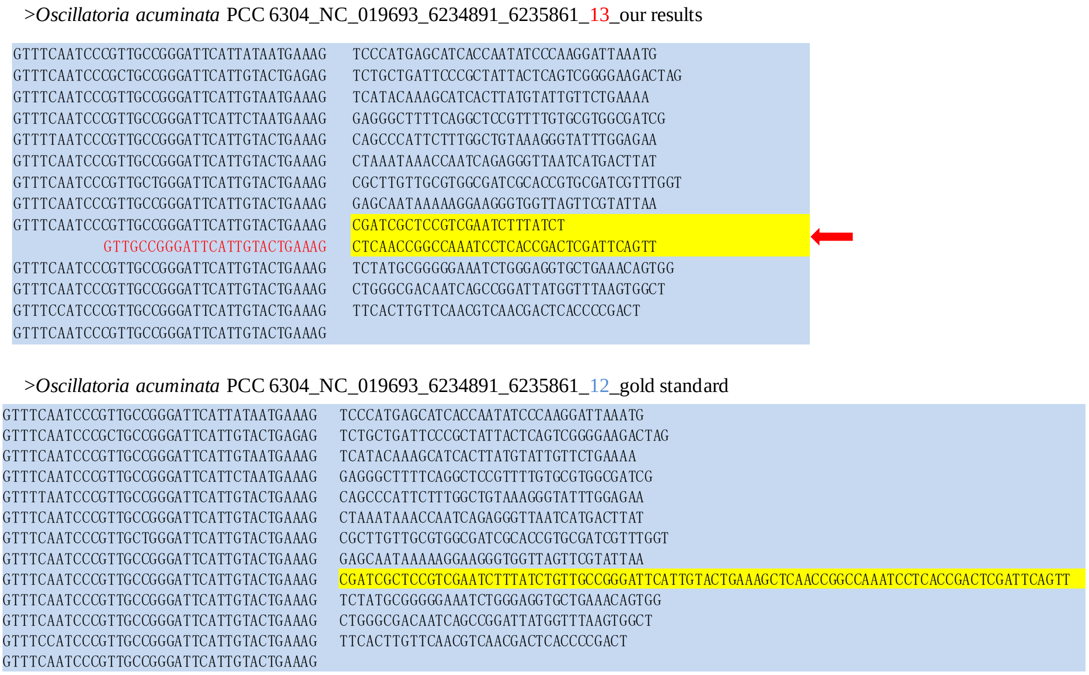

> *Chlorobium phaeobacteroides* DSM 266_NC_008639_1625359_1633049_115_our results

GTTTCAATCCACGCGCCCGCAGGGGGCGCGAC	AGGCTATCCGTATCTGCGCTTTCACGTCTGTAAGCT
GTTTCAATCCACGCGCCCGCAGGGGGCGCGAC	CTGTCATTGATGGCCGATGATTTCCGTGAAATAG
GTTTCAATCCACGCGCCCGCAGGGGGCGCGAC	AAGGCCAAATCGACCACATCGACGGCGACGGTCT
GTTTCAATCCACGCGCCCGCAGGGGGCGCGAC	GTGATGTCGCGTTCCGGCACGACGCGCTCAAAAA
GTTTCAATCCACGCGCCCGCAGGGGGCGCGAC	CTGCATCCACGGCAGTTCAGGGGAGGGATTCGGTTAT
GTTTCAATCCACGCTCCCGC AGGGGGCGCGAC	GGTGTCACTTACGGTTGTGTTTTTTGATCGGAATCGA
TTCAATCCACGCCGCCCGCCAGGGGGCGCTAC	CATTGAATACTACAAAGATGGAACCTCAACTACTC
GTTTCAATCCACGCGCCCGCAGGGGGCGCGAC	CATGCTTGCCATTACCCTGATCGCAAGCCACCCAA
GTTTCAATCCACGCGCCCGCAGGGGGCGCGAC	AAAAGGTGATTGTGATCACACACCTGCCAAGCAT
GTTTCAATCCACGCGCCCGCAGGGGGCGCGAC	TCGGATTGTACTCTCTTGCCAAGTGGGTGATCGGCA
GTTTCAATCCACGCGCCCGCAGGGGGCGCGAC	AGTTGTCCAGGCCTTGCACGACCTCAATCAACGG
GTTTCAATCCACGCGCCCGCAGGGGGCGCGAC	AGGCTATCCGTATCTGCGCTTTCACGTCTGTAAGCT
………	
GTTTCAATCCACGCGCCCGCAGGGGGCGCGAC	GCATGGTCGAGAAAGAGTTGCCTGAATCGGCACCTGT
GTTTCAATCCACGCGCCCGCAGGGGGCGCGAC	CAATGCTTCTGTTAAGTTGAGCAAGCGCAATCACTG
GTTTCAATCCACGCGCCCGCAGGGGGCGCGAC	CACAGCCCGCCCTTATCAGCAGGCGTTGTTCGAGGC
GTTTCAATCCACGCGCCCGCAGGGGGCGCGAC	

> *Chlorobium phaeobacteroides* DSM 266_NC_008639_1625359_1633049_114 _gold standard

GTTTCAATCCACGCGCCCGCAGGGGGCGCGAC	AGGCTATCCGTATCTGCGCTTTCACGTCTGTAAGCT
GTTTCAATCCACGCGCCCGCAGGGGGCGCGAC	CTGTCATTGATGGCCGATGATTTCCGTGAAATAG
GTTTCAATCCACGCGCCCGCAGGGGGCGCGAC	AAGGCCAAATCGACCACATCGACGGCGACGGTCT
GTTTCAATCCACGCGCCCGCAGGGGGCGCGAC	GTGATGTCGCGTTCCGGCACGACGCGCTCAAAAA
GTTTCAATCCACGCGCCCGCAGGGGGCGCGAC	CTGCATCCACGGCAGTTCAGGGGAGGGATTCGGTTAT
GTTTCAATCCACGCTCCCGCAGGGGGCGCGAC	GGTGTCACTTACGGTTGTGTTTTTTGATCGGAATCGATTCAATCCACGCCGCCCGCCAGGGGGCGCTACCATTGAATACTACAAAGATGGAACCTCAACTACTC
GTTTCAATCCACGCGCCCGCAGGGGGCGCGAC	CATGCTTGCCATTACCCTGATCGCAAGCCACCCAA
GTTTCAATCCACGCGCCCGCAGGGGGCGCGAC	AAAAGGTGATTGTGATCACACACCTGCCAAGCAT
GTTTCAATCCACGCGCCCGCAGGGGGCGCGAC	TCGGATTGTACTCTCTTGCCAAGTGGGTGATCGGCA
GTTTCAATCCACGCGCCCGCAGGGGGCGCGAC	AGTTGTCCAGGCCTTGCACGACCTCAATCAACGG
GTTTCAATCCACGCGCCCGCAGGGGGCGCGAC	CTCGTGATCAAGGGTATGCAGGTGAGCGTAGACATC
GTTTCAATCCACGCGCCCGCAGGGGGCGCGAC	AGGCTATCCGTATCTGCGCTTTCACGTCTGTAAGCT
………	
GTTTCAATCCACGCGCCCGCAGGGGGCGCGAC	GCATGGTCGAGAAAGAGTTGCCTGAATCGGCACCTGT
GTTTCAATCCACGCGCCCGCAGGGGGCGCGAC	CAATGCTTCTGTTAAGTTGAGCAAGCGCAATCACTG
GTTTCAATCCACGCGCCCGCAGGGGGCGCGAC	CACAGCCCGCCCTTATCAGCAGGCGTTGTTCGAGGC
GTTTCAATCCACGCGCCCGCAGGGGGCGCGAC	

>*Frankia* sp. CcI3_NC_007777_3904715_3905896_16_our results

CTTGGGAACCTCGCCGGGCGCGGGCGCCCGGCGCTGCT	CTTCTGGGTCGCGATATGGTGCCTTATCGTGACTC
GTTGTGATCCTCGGCGCTGCCCTCGCGGCAGATCCAGC	GCCCAGTTCGCGCATCTTCGC
GTTGCGATCCTCGCCGAGGGCGGTCCCTCGGCGCTGC	TGGTGGGCGAGGTGGGCCTGCGCCAGCGTCAGGTT
GTTGTGATCCTCGCCGAGGGCGGTCCCTCGGCGCTGC	GACGACCTGGTGCTGATCTGCACCAACGGCACCGCC
GTTGTGATCCTCGCCGAGGGCGATCCCTCGGCGCTGC	CGTCCGCCGCGACAGCAAAGACCTACACGCCACCCACC
GTTGTGATCCTCGCCGAGGGCGATCCCTCGGCGCTGC	GTGTCCGCCTCCCGTGAGCTGCTCGTCCGCTGGTT
GTTGTGATCCTCGCCGAGGGCGATCCCTCGGCGCTGC	CTGGGACACGGCCCGGATCCTCGCCGAGCGCAACGCC
GTTGTGATCCTCGCCGAGGGCGATCCCTCGGCGCTGC	GATGGTGACGACCTGGTGCTGATCTGCACCAACG
GTTGTGATCCTCGCCGAGGGCGATCCCTCGGCGCTGC	TCGATGGTGCCGTCGGGCTGGTCAACGCCTTGGAT
GTTGTGATCCTCGCCGAGGGCGATCCCTCGGCGCTGC	AAGTGCTCGAACATGTCGTCGACTGCATCCGGGT
GTTGTGATCCTCGCCGAGGGCGATCCCTCGGCGCTGC	CCCCGGCCGGCGCTCACCGTCGCCGCCTGGTCCTAT
GTTGTGATCCTCGCCGAGGGCGATCCCTCGGCGCTGC	TCGCGAACACCAGCTGGTTGGAGCCACGCGGGTA
GTTGTGATCCTCGCCGAGGGCGATCCCTCGGCGCTGC	TGCGCCCCCGCTATCTGGTCCCGCTTTTCCTCGGGA
GTTGTGATCCTCGCCGAGGGCGATCCCTCGGCGCTGC	GACCGCTGCTTGAGCCGACCGGTCACAATGACCC
GTTGTGATCCTCGCCGAGGGCGATCCCTCGGCGCTGC	CCGCTCCGGCCTGGATCGCCAGATGTCCGCCCTTGCG
GTTGTGATCCTCGCCGAGGGCGATCCCTCGGCGCTGC	CTGTAGGGGCACGTTGTAATGCTTTCCGGCCCGGAG
GTTGTGATCCTCGCCGAGGGCGATCCCTCGGCGCTGC	

>*Frankia* sp. CcI3_NC_007777_3904716_3905896_15_gold standard

CTTGGGAACCTCGCCGGGCGCGGGCGCCCGGCGCTGC	TTCTGGGTCGCGATATGGTGCCTTATCGTGACTCAGTTGTGATCCTCGGCGCTGCCCTCGCGGCAGATCCAGCCCAGTTCGCGCATCTTCGC
GTTGCGATCCTCGCCGAGGGCGGTCCCTCGGCGCTGC	TGGTGGGCGAGGTGGGCCTGCGCCAGCGTCAGGTT
GTTGTGATCCTCGCCGAGGGCGGTCCCTCGGCGCTGC	GACGACCTGGTGCTGATCTGCACCAACGGCACCGCC
GTTGTGATCCTCGCCGAGGGCGATCCCTCGGCGCTGC	CGTCCGCCGCGACAGCAAAGACCTACACGCCACCCACC
GTTGTGATCCTCGCCGAGGGCGATCCCTCGGCGCTGC	GTGTCCGCCTCCCGTGAGCTGCTCGTCCGCTGGTT
GTTGTGATCCTCGCCGAGGGCGATCCCTCGGCGCTGC	CTGGGACACGGCCCGGATCCTCGCCGAGCGCAACGCC
GTTGTGATCCTCGCCGAGGGCGATCCCTCGGCGCTGC	GATGGTGACGACCTGGTGCTGATCTGCACCAACG
GTTGTGATCCTCGCCGAGGGCGATCCCTCGGCGCTGC	TCGATGGTGCCGTCGGGCTGGTCAACGCCTTGGAT
GTTGTGATCCTCGCCGAGGGCGATCCCTCGGCGCTGC	AAGTGCTCGAACATGTCGTCGACTGCATCCGGGT
GTTGTGATCCTCGCCGAGGGCGATCCCTCGGCGCTGC	CCCCGGCCGGCGCTCACCGTCGCCGCCTGGTCCTAT
GTTGTGATCCTCGCCGAGGGCGATCCCTCGGCGCTGC	TCGCGAACACCAGCTGGTTGGAGCCACGCGGGTA
GTTGTGATCCTCGCCGAGGGCGATCCCTCGGCGCTGC	TGCGCCCCCGCTATCTGGTCCCGCTTTTCCTCGGGA
GTTGTGATCCTCGCCGAGGGCGATCCCTCGGCGCTGC	GACCGCTGCTTGAGCCGACCGGTCACAATGACCC
GTTGTGATCCTCGCCGAGGGCGATCCCTCGGCGCTGC	CCGCTCCGGCCTGGATCGCCAGATGTCCGCCCTTGCG
GTTGTGATCCTCGCCGAGGGCGATCCCTCGGCGCTGC	CTGTAGGGGCACGTTGTAATGCTTTCCGGCCCGGAG
GTTGTGATCCTCGCCGAGGGCGATCCCTCGGCGCTGC	

Figure S5. 59 cases with DRs broken by a transposon

(a) CRISPRs with DRs inserted by transposons in the *Microcystis aeruginosa* NIES-843. DRs and spacers are represented in the left and right columns, respectively. The newly annotated CRISPR regions are in red, and the new spacers are marked by yellow background. The last number in the CRISPR name is the DR copy number. **(b)~(k)** The other CRISPRs with DRs inserted by transposons.

```
GTTCCAATTAATCTTAAACCCTATTAGGGATTGAAAC  TTCAGAATTCGACACAATTATTGCTATAAAAAATGATAGC
.........
GTTCCAATTAATCTTAAACCCTATTAGGGATTGAAAC  TTGTAATTGAGCTTCGTTGTACAGCCAATTTAATC
GTTCCAATTAATCTTAAACCCTATT              MITE(180bp)
                     CTATTAGGGATTGAAAC  TCCTGCTTTGGTAAAATTACTGATGCCCATAAGC
GTTCCAATTAATCTTAAACCCTATTAGGGATTGAAAC  CAGCCCGAATGGTACGAGACAGCCTGGGCAGACCAA
.........
GTTCCAATTAATCTTAAACCCTATTAGGGATTGAAAC  TTCTTTTGATGATGATGCTAACCGGGAAGCAATC
GTTCCAATTAATCTTAAACCCTATTAGGGA         transposase (513bp)-transposase (675bp)-
                                       MITE(167bp)
                        TTAGGGATTGAAAC  AGCAATATTTTTGGCATCGGATTCGGTCAAAACT
GTTCCAATTAATCTTAAACCCTATTAGGGATTGAAAC  CTTAAGACAGTGCCTTTGAGCGATAAGGGCGATTATC
.........
GTTCCAATTAATCTTAAACCCTATTAGGGATTGAAAC  CTCACTTTAGGCTAGTGTTTGCCCTCTCGTGAAAAAGCAA
GTTCCAATTAATCTTAAACCCTATTAGGGATTGAA
```

Microcystis aeruginosa NIES-843_NC_010296_ _2814769_2822874_112_our results
Microcystis aeruginosa NIES-843_NC_010296_ _2814769_2822884_112 _gold standard

Microcystis aeruginosa NIES-843_NC_010296_ _2823052_2824590_21 _our results
Microcystis aeruginosa NIES-843_NC_010296_ _2823103_2824595_20_gold standard

Microcystis aeruginosa NIES-843_NC_010296_ _2826180_2829164_41_our results
Microcystis aeruginosa NIES-843_NC_010296_ _2826228_2829166_40_gold standard

GGGACTTAGGCATTTTCGGGCAGCAAAAAAGGCTCAAACCATTACGGGACAAAGGGTTAACCTCGATTTATGATTTTCCTTGATCTATCTGGGTTTCAGCGATTTTG GGCTTCTCGAAGTAAATCCCAAGCAGGGATTGG GGTTGAGGCTTTTCTCGATTTGTTTTTTGTCTAAGTCCCA (180bp, appears 19 times)

TTGTTATACTCGAAAAAGCTCGGAAGTATAAGAGTTTGTGCTGCCTATTTTTTCGGAAAAATAGGTTGTAATGGGAGACTCTTTGTTTTCCTAGACAAAAATAATTACT TTTTCAAAAAAACACTTTTCTATTTTTTTGTTGGGTATTTTATTCTCTGGAAGTCCCT(167bp, appears 8 times)

```
                                 Transposase (1080bp)- Transposase (426bp)
 CACTATTTTCAGGATAGGTAGGCTAAAA    AAACGAGGTAGGTATCAAGGCGGGTGTATCTGCCGCTGATAA
GTTTCAATTCCTCATAGGTAGGCTAAAAAC  CCCAATCCGAAATTAGAGGTAGGAGAAATGCAGGAGT
.........
GTTTCAATTCCTCATAGGTAGGCTAAAAAC  CATCATTGAGCCGATTTTTGAGTCTCGGTTTATTTA
GTTTCAATTCCTCATAG               Transposase (1221bp)
              ATAGGTAGGCTAAAAAC  CCCGCTTGCACTAGGATATATTTTCGGCGAGGCAGCGGT
GTTTCAATTCCTCATAGGTAGGCTAAAAAC  GAAGATGCAATAAGAGTAGCAGATAAAATGCTGAA
.........
GTTTCAATTCCTCATAGGTAGGCTAAAAAC  TCTTTCCTATTCTGTTTTAATACTATAGAACTAAT
GTTTCAATTCCTCATAGGTAGGCTAAAAAC
```

Thermoanaerobacter italicus Ab9_NC_013921_ _2330986_2331339_5_our results
Thermoanaerobacter italicus Ab9_NC_013921_ _2330986_2331286_4_gold standard

Thermoanaerobacter italicus Ab9_NC_013921_ _2332916_2334204_19 _our results
Thermoanaerobacter italicus Ab9_NC_013921_ _2332916_2334204_19 _gold standard

```
GTCAGAACGACTTCCCTGATGAAGAAGGGATTAAGAC  GCCGCCGCCGCTCTTGATGCCGCGCATGTACTG
.........
GTCAGAACGACTTCCCTGATGAAGAAGGGATTAAGAC  GGCTGGCCGCGTCCATGGCATCCATGTCACGGC
GTCAGAACGACTTCCCTGATGAAGA              IS66 (927bp)-phage DNA methylase (641bp)
                GATGAAGAAGGGATTAAGAC  CTTCATCAGAATGGCGCCTCGTCGTCATCGGTGGG
GTCAGAACGACTTCCCTGATGAAGAAGGGATTAAGAC  TGCCCTGACGGACGCTGTTGAGGTTCTGCAG
.........
GTCAGAACGACTTCCCTGATGAAGAAGGGATTAAGAC  TTCGCAATTTCTGAGGATCATTCGTGTTCTTCGT
GTCAGAACGACTTCCCTGATGAAGAAGGGATTAAGAC
..................
TTTCTGAGCTGCCTGCGCGGCAGCGAAC           CAGGGATAGGTGATGGTTCTATTACGGTTATG
.........
TTTCTGAGCTGCCTGCGCGGCAGCGAAC           ATCATCGAGGAAGCCGCCAAGGTCGGCAGCCA
TTTCTGAGCTGCCTGCGCGGCAGCGAAC           ISPsy4 (222bp)- istB(171bp)
                CGCGGCAGCGAAC           CCCTCAGATATTCGCCGGCGGTGATGATCTGGAT
TTTCTGAGCTGCCTGCGCGGCAGCGAAC           CTGGCCCAAGCCGACGACCCCGGCTGCTACGA
.........                              IS110(1383bp)
TTTCTGAGCTGCCTGCGCGGCAGCGAAC           GGCGGCAGTCCGGTGGTCGCGATTTTGGATGA
TTTCTGAGCTGCCTGCGCGGCAGCGAAC
TTTCTGAGCTGCCTGCGCGGCAGCGAAC           GCTGGCGGGAACCTCATGAGCATGGCTGGCGA
.........
TTTCTGAGCTGCCTACGCGGCAGCGAAC           GGGGCGATCAGGTCGGGGGGTCGAGGGATCGA
TTTCTGAGCTGCCTGCGCGGCAGCGAAC
```

Thioalkalivibrio nitratireducens DSM 14787_NC_019902 _1466441_1467521_15 _our results
Thioalkalivibrio nitratireducens DSM 14787_NC_019902_ _1466441_1467533_15 _gold standard

Thioalkalivibrio nitratireducens DSM 14787_NC_019902 _1469911_1471052_16 _our results
Thioalkalivibrio nitratireducens DSM 14787_NC_019902 _1469966_1471052_15 _gold standard

Thioalkalivibrio nitratireducens DSM 14787_NC_019902 _2520192_2523887_61 _our results
Thioalkalivibrio nitratireducens DSM 14787_NC_019902 _2520192_2523887_61 _gold standard

Thioalkalivibrio nitratireducens DSM 14787_NC_019902 _2928156_2928605_7 _our results
Thioalkalivibrio nitratireducens DSM 14787_NC_019902 _2928217_2928605_6 _gold standard

Thioalkalivibrio nitratireducens DSM 14787_NC_019902 _2931057_2932046_16 _our results
Thioalkalivibrio nitratireducens DSM 14787_NC_019902 _2931057_2932046_16 _gold standard

GTTTCAATTCCTCATAGGTACGCTGAGAAC	GCATTGGCTTTCATCTCCCCCTCGGCATGCCCGGC
....	
GTTTCAATTCCTCATAGGTACGCTGATAAC	GCTCTGGATGCTTGGAAGAAATGCAAGGAACGGAATA
GTTTCAATTCCTCATAG	IS110 (2800bp)
AGGTACGCTGAGAAC	ACATAATATTCCTTGTATGCATAAACCACGCCGTCAGGA
GTTTCAATTCCTCATAGGTACGCTGAGAAC	TCTGCACATTGGGGGCATAAAGCGACGCCCCCAAAACC
....	
GTTTCAATTCCTCATAGGTACGCTGAAAAC	TTCTCGGCTGGCAGGCTGATTTAGAGGAGGTGTTCTAA
GTTTCAATTCCTCATA	IS1634(1701bp)
TCATAGGTACGCTGAAAAC	CGGTAGATAGTGCCGACGAGGCTCTT
GTTTCAATTCCTCATAGGTACGCTGAAAAC	CGATATTGTTCAAACTTTGCATTCATTTCGTTCCT
....	
GTTTCAATTCCTCATAGGTACGCTGAAAAC	GAAATATACCATGCGGGGGTGTTTCAGTTCCGGTTCA
GTTTCAATTCCTCATAG	IS110 (1215bp)
AGGTACGCTGAAAAC	TAAAACTCGTATTCCTTCCCAGTCTTTTTCTTTGGGCA
GTTTCAATTCCTCATAGGTACGCTGAAAAC	ACGGAACCACTCCTGCCAATATGCTTCTTTTTCGTTTC
....	
GTTTCAATTCCTCATAGGTACGCTGAAAAC	CCCGGACAATACCCTCATGATACCGTCCTCGGTTCTTC
GTTTCAATTCCTCATAGGTACGCTGAAAAC	
....	
GTTTCAATTCCTCATAGGTACGCTGAAAAC	TTTGACCACCACCTCATCCTTAGCAAATGCTAACGGA
....	
GTTTCAATTCCTCATAGGTACGCTGAAAAC	ACATTTTCAATTTTATGTAGATTGAAATATCGTTCTG
GTTTCAATTCCTCATAG	MITE (300bp)
AGGTACGCTGAAAAC	CGGTGTGTGATTGTATGAAAGATGACGATGACCAGGGC
GTTTCAATTCCTCATAGGTACGCTGAAAAC	ATGTGCCGGAGGTGTGCCCGAACTGCGCAGATATCC
....	
GTTTCAATTCCTCATAGGTACGCTGAAAAC	GCATGGACTGTGAGCGGATGTACGTGCTCTGGGAGTA
GTTTCAATTCCTCATA	IS1634(1701bp)
TCATAGGTACGCTGAAAAC	GCCGAGAAAATGTTCTCGGTGGCCTGGGATTACGCT
GTTTCAATTCCTCATAGGTACGCTGAAAAC	GATGCCCTCCTTTCTTGCGATTGAACGGCAAGAAGTG
....	
GTTTCAATTCCTCATAGGTACGCTGAAAAC	CGGACCGCTTCAACCCCAGGCAGGAGGTTTTGACCTG
GTTTCAATTCCTCATAG	MITE (300bp)
AGGTACGCTGAAAAC	GACTGGCAGAGATTCGGGATGTCGTGAGGCAGTATC
....	
GTTTCAATTCCTCATAGGTACGCTGAAAAC	GAGGGAGAATGCCGCATGGTCGGGTCGAGTTCCGCGG
GTTTCAATTCCTCATAGGTACGCTGAAAAC	

Thermacetogenium phaeum DSM 12270_NC_018870_ _2439391_2440133_11_our results
Thermacetogenium phaeum DSM 12270_NC_018870_ _2439391_2440146_11_gold standard

Thermacetogenium phaeum DSM 12270_NC_018870_ _2443573_2445992_36_our results
Thermacetogenium phaeum DSM 12270_NC_018870_ _2443641_2446006_35_gold standard

Thermacetogenium phaeum DSM 12270_NC_018870_ _ 2447892_2448160_4 _our results
Thermacetogenium phaeum DSM 12270_NC_018870_ _ 2447944_2448173_3_gold standard

Thermacetogenium phaeum DSM 12270_NC_018870_ _ 2449719_2451060_20_our results
Thermacetogenium phaeum DSM 12270_NC_018870_ _ 2449704_2451060_20_gold standard

Thermacetogenium phaeum DSM 12270_NC_018870_ _2453564_2455390_27_our results
Thermacetogenium phaeum DSM 12270_NC_018870_ _2453564_2455337_26_gold standard

Thermacetogenium phaeum DSM 12270_NC_018870_ _ 2455691_2458143_37_our results
Thermacetogenium phaeum DSM 12270_NC_018870_ _ 2455676_2458157_37_gold standard

Thermacetogenium phaeum DSM 12270_NC_018870_ _2460043_2463456_51_our results
Thermacetogenium phaeum DSM 12270_NC_018870_ _2460106_2463402_49_gold standard

Thermacetogenium phaeum DSM 12270_NC_018870_ _ 2463757_2464503_11_our results
Thermacetogenium phaeum DSM 12270_NC_018870_ _ 2463742_2464503_11_gold standard

```
GTCGTAATCCCTTCAAA                                MITE(49bp)
              AAATCAGGTCATTAATTCCAAT    TGAAGATTAATGACCCGAAGCAAATCCAGATTTTAGAAGA
GTCGTAATCCCTTCAAATCAGGTCATTAATTCCAAT    CATAGGCTCATTAAAGGGCATGTAAGCATGGAGCTTGT
.........
GTCGTAATCCCTTCAAATCAGGTCATTAATTCCAAT    GGAAGGGGCAACAACCCTTCCGGAGGGTTTTAAAATGA
GTCGTAATCCCTTCAAATCAGGTCATTAATTCCAAT
.................                        IS4 (1368bp)
```

Flexistipes sinusarabici DSM 4947_NC_015672_ _1428433_1430685_30 _our results

Flexistipes sinusarabici DSM 4947_NC_015672_ _1428495_1430685_29_gold standard

```
GTCGTAATCCCTTCAAA                                MITE(49bp)
              AAATCAGGTCATTAATTCCAAT    AGTATATCTTAACTTAACAAATGGATTAGAAGACTTTT
GTCGTAATCCCTTCAAATCAGGTCATTAATTCCAAT    TCGGGTGCTGCGTATTATGCAACTGCTCCCACGGAAGGG
GTCGTAATCCCTTCTAATCAGGTCAATTCC
.................
```

Flexistipes sinusarabici DSM 4947_NC_015672_ _1709402_1709566_2 _our results

Flexistipes sinusarabici DSM 4947_NC_015672 _1709462_1709573_1 _gold standard

```
GTTGTAATCCCTTCAAA                                MITE(47bp)
              AAATCAGGTCATGGATTCCAAT    GGTATAGATAATAAAAAAATTGTCCATGTATTTGATATA
GTCGTAATCCCTTCTAATCAGGTCATTAATTCCAAT    AATAAACTATCCAAGAACTTTTGGAATTGAGATAGAA
.........
GTCGTAATCCCTTCAAGTCAGGTCATGAATTCCAAT    AAATTTCCGGACTGAGGCAATTAGGATGGCAAAAAAA
GTCGTAATCCCTTCTAATCAGGTCAATTCC
.................
```

Flexistipes sinusarabici DSM 4947_NC_015672_ _1709819_1710226_5 _our results

Flexistipes sinusarabici DSM 4947_NC_015672 _1709819_1710226_5 _gold standard

```
GTTGTAATCCCTTCAAA                                MITE(47bp)
              AAATCAGGTCATGGATTCCAAT    GTTGAAGCACTCGGCGCAGTTCTATCTTTTGAGCAGA
GTCGTAATCCCTTCTAATCAGGTCATGGATTCCAAT    AGAGAGATTTCTCCATTTCCTAAATTTAATACAGTTGAT
.........
GTCGTAATCCCTTCTAATCAGGTCATGGATTCCAAT    TACACAACTAAAACAGATACTTTGATTATTGTCTTTTCCTA
GTCGTAATCCCTTCTAATCAGGTCAATTCC
.................
```

Flexistipes sinusarabici DSM 4947_NC_015672_ _1710472_1710885_5 _our results

Flexistipes sinusarabici DSM 4947_NC_015672_ _1710472_1710885_5 _gold standard

```
GTTGTAATCCCTTCAAA                                MITE(47bp)
              AAATCAGGTCATGGATTCCAAT    CTGAAGCGAATCAATTATATAAAAATTCTTTCTCTTCT
GTCGTAATCCCTTCTAATCAGGTCATGGATTCCAAT    AGTGATGCGGGGTGGGTACGTCGGTTTGCATATCGAAA
.........
GTCGTAATCCCTTCTAATCAGGTCATGGATTCCAAT    GCCGGAAGGGAGCACAACCCTTCCGGAGGGTTACAAAA
GTCGTAATCCCTTCTAATCAGGTCATGGATTCCAAT
.................
```

Flexistipes sinusarabici DSM 4947_NC_015672_ _1711131_1712652_20_our results

Flexistipes sinusarabici DSM 4947_NC_015672_ _1711131_1712652_20_gold standard

```
GTTGTAATCCCTTCAAA                                MITE(47bp)
              AAGTCAGGTCATGAATTCCAAT    TGGGAAATATATAGATACCTGTCCTTCCGGGTTTGA
GTCGTAATCCCTTCAAGTCAGGTCATGAATTCCAAT    TTAAAAAGAATCCCAAAGAAAATAGAGGAACTCCTCAAG
.........
   GCAATCCCTTCAAGTCAGGTCATGAATTCCAAT    ATCTTCGTCCTCGTGGGATATCCCCGAGGACTTGAAAAAA
GTCGTAATCCCTTCAAGTCAGGTCATGAATTCCAAT
```

Flexistipes sinusarabici DSM 4947_NC_015672_ _1734298_1735756_19 _our results

Flexistipes sinusarabici DSM 4947_NC_015672_ _1734298_1735756_19 _gold standard

ACATTTGCGCAATATCTCATCGCAAATGTTTCTGTTCAGAGCTTGCTGG（49bp， appears 3 times ）

ACCCAGCCATCATATAATCGGCTGGGTTTCTGGTCCGAGCTTGCTGG（47bp， appears 4 times ）

GTTTCAATTACCGTAGGTACTATCAGAAC	TCATATAGGTGCATTCCTTCATTCGACATAATCCGCAAA
TTTCAAAGCCTTAAAGGTACTATCAGAAC	TTGGACAAGCTAAAGCTAGGGGCTTTACGGCGACATCCTA
………	
GTTTCAATTCCTTATAGGTACTATCAGAAC	CGATACGGGCGATGTCATAGTGATACTTCGCCCAGATGAGA
TTCAATTCCTTATAGGTACTATCAGAAC	IS110(1105bp)
GTTTCAATTCCTCATAGGTACGATCAAAAC	TTCACACTTCGGCATGTCGCGCCCCTCGGACTTACGAC
………	
GTTTCAATTCCTCATAGGTACGATCAAAAC	TATAGTGCTCGGGTTCAAGGACGCCGGAATATACCGG
GTTTCAATTCCTCATAG	IS110(1227bp)
ATAGGTACGATCAAAAC	GGAGCCTAAACCATGCGTCAATATCGCGGACGAAT
GTTTCAATTCCTCATAGGTACGATCAAAAC	GAGCAGCCGGCGCCGCAATCCGATGCACAGACGCAG
………	
GTTTCAATTCCTCATAGGTACGATCAAAAC	ACTCCTGCAGCGCCTCGAGGCGCGCCAGCCGACTTT
GTTTCAATTCCTCATAG	IS110(1227bp)
ATAGGTACGATCAAAAC	CATCAGCGCGCCGACAACGGCGCCCACGTCGACACCG
GTTTCAATTCCTCATAGGTACGATCAAAAC	GCAAACGGAACGCGTGCCGGCGCCGCCGTGGTTTGT
………	
GTTTCAATTCCTCATAGGTACGATCAAAAC	CGGGACGATCTATGTCATGTTCTCGGCGGTGGAAGT
GTTTCAATTCCTCATAG	IS110(1227bp)
ATAGGTACGATCAAAAC	AAAGGAATGGGCAGACATCGTGCTGTTCCTGAATTA
GTTTCAATTCCTCATAGGTACGATCAAAAC	CATGTCGAGCTCCACCATGGAGAGCCCGATCCGCCGCGC
………	
GTTTCAATTCCTCATAGGTACGATCAAAAC	CTGTATCGTATATTCAGTTCCTGATCCTGCGAAGCATT
GTTTCAATTCCTCATAG	IS110(1228bp)
ATAGGTACGATCAAAAC	GTTACGGCTGTGCTCCACTAGCCGCTTCCACGCTTC
GTTTCAATTCCTCATAGGTACGATCAAAAC	GTGATACTGTCCGAATACTTCGTGACCGTCATATCCGT
………	
GTTTCAATTCCTCATAGGTACGATCAAAAC	GTGAGCCATCAGAAATATCTCTCGTTCCCTATCCGT
GTTTCAATTCCTCATAGGTACGATCAAAAC	
………………	IS(1293bp)-IS(1278bp)-IS110 (1224bp)
ATAGGTACGATCAAAAC	GATCGAAAAGCGAGAAACGATTGAGTTGAACGTTGACGA
GTTTCAATTCCTCATAGGTACGATCAAAAC	GAAGGAGTTTACCGTCGCCGTCATAACGCTGGAGT
………	
GTTTCAATTCCTCATAGGTACGATCAAAAC	TGAGGTGGTTGTGAATTATAGAAGCCTGAAAACAGG
GTTTCAATTCCTCATAG	IS110(1227bp)
ATAGGTAAGATCAAA	AAACCTACATCCGCATGGACTACACTGGTACAGATCGCTAC
GTTTCAATTCCTCATAGGTACGATCAAAAC	GATGCCCTGAAGTTCCCAGACGGCCTGATACGCCA
………	
GTTTCAATTCCTCATAGGTACGATCAAAAC	TCAGTTTGGCCATGTATGTGATCTTCGGCATTTCGT
GTTTCAATTCCTCATAG	IS110(1225bp)
ATAGGTACGATCAAAAC	ACCGATGCCGAGAACATGGAGCTAAGAATCCGTATG
GTTTCAATTCCTCATAGGTACGATCAAAAC	TTTGCATGTACATGGGCAGGCGCGGAACAGAGATCG
GTTTCAATTCCTCATAGGTACGATCAAAAC	GGCCACGATCTCACCCACGCTGTCGTCACCGGTGT
GTTTCAATTCCTCATAGGTACGATCAAAAC	

Thermobacillus composti KWC4_NC_019897_ _3270701_3271073_5 _our results
Thermobacillus composti KWC4_NC_019897_ _3270768_3271074_4 _gold standard

Thermobacillus composti KWC4_NC_019897_ _3272406_3273774_20_our results
Thermobacillus composti KWC4_NC_019897_ _3272406_3273720_19 _gold standard

Thermobacillus composti KWC4_NC_019897_ _3275324_3277594_34 _our results
Thermobacillus composti KWC4_NC_019897_ _3275311_3277541_33_gold standard

Thermobacillus composti KWC4_NC_019897_ _3279144_3280208_16 _our results
Thermobacillus composti KWC4_NC_019897_ _3279131_3280155_15 _gold standard

Thermobacillus composti KWC4_NC_019897_ _3281758_3283032_19 _our results
Thermobacillus composti KWC4_NC_019897_ _3281745_3282977_18 _gold standard

Thermobacillus composti KWC4_NC_019897_ _3284583_3285001_6_our results
Thermobacillus composti KWC4_NC_019897_ _3284570_3285001_6 _gold standard

Thermobacillus composti KWC4_NC_019897_ _3298570_3299110_8_our results
Thermobacillus composti KWC4_NC_019897_ _3298557_3299057_7_gold standard

Thermobacillus composti KWC4_NC_019897_ _3300646_3300996_5_our results
Thermobacillus composti KWC4_NC_019897_ _3300647_3300943_4_gold standard

Thermobacillus composti KWC4_NC_019897_ _3302548_3302761_3_our results
Thermobacillus composti KWC4_NC_019897_ _3302535_3302761_3_gold standard

GTTTTTAGCCTACCTATAAGGAATTGAAAC	AGCTGAAAGACATTTTAAAGTAGCAGGGCAAGGTACAG
………	
GTTTTTAGCCTACCTATAAGGAATTGAAAC	AGTATTAAAAATTAAGGAGGTTTAAAAAATGGAAAA
GTTTTTAGCCTACCTAT	IS110 (1221bp)
CTATAAGGAATTGAAAC	CTAATTTGCTTTAAGCTTGTTGCTACAAGATAGACCTTG
GTTTTTAGCCTACCTATAAGGAATTGAAAC	CGCCGAGGTCTTCTCCTGTCGCGATATAATGCGACAGG
………	
GTTTTTAGCCTACCTATAAGGAATTGAAAC	AGAGGAACAATTGGAATGTATGAAATAGGACAAAGA
GTTTTTAGCCTACCTAT	IS1634 (2879bp)
CTATAAGGAATTGAAAC	AGGTCAAACCTGGGGCAAAGACTTACGACGGGAAA
GTTTTTAGCCTACCTATAAGGAATTGAAAC	TGCCATCACCTATCATACTATCTCCATCCACTCTTA
………	
GTTTTTAGCCTACCTATAAGGAATTGAAAC	TGGATAGCAAAGGCAAGTGCAGAACTGGATAAAAAGA
GTTTTTAGCCTACCTAT	IS110 (1221bp)
CTATAAGGAATTGAAAC	CCTATCAGCTCCGGTGGCACCCCAAACGCAATAGC
GTTTTTAGCCTACCTATAAGGAATTGAAAC	GATAGCCTTCAGCTATTAACTTTGCCGCTTCCAATTG
………	
GTTTTTAGCCTACCTATAAGGAATTGAAAC	CCATGTTGGTTTTACGCTCAATCTCGGTTACGTAGAAA
GTTTTTAGCCTACCTAT	IS110 (1221bp)

Thermoanaerobacter brockii subsp. finnii Ako-1_ NC_014964_121660_124897_48 _our results
Thermoanaerobacter brockii subsp. finnii Ako-1_ NC_014964_121660_124897_48 _gold standard

Thermoanaerobacter brockii subsp. finnii Ako-1_NC_014964_126473_128016_23 _our results
Thermoanaerobacter brockii subsp. finnii Ako-1_ NC_014964_126529_128029_22 _gold standard

Thermoanaerobacter brockii subsp. finnii Ako-1_NC_014964_131265_133542_34_our results
Thermoanaerobacter brockii subsp. finnii Ako-1_NC_014964_131317_133555_33_gold standard

Thermoanaerobacter brockii subsp. finnii Ako-1_NC_014964_135132_136869_26 _our results
Thermoanaerobacter brockii subsp. finnii Ako-1_NC_014964_135184_136882_25_gold standard

GTTTTTAGCCTACCTATAAGGAATTGAAAC	AGCTGAAAGACATTTTAAAGTAGCAGGGCAAGGTACAG
....	
GTTTTTAGCCTACCTATAAGGAATTGAAAC	AGTATTAAAAATTAAGGAGGTTTAAAAAATGGAAAA
GTTTTTAGCCTACCTAT	IS110 (1221bp)
CTATAAGGAATTGAAAC	CTAATTTGCTTTAAGCTTGTTGCTACAAGATAGACCTTG
GTTTTTAGCCTACCTATAAGGAATTGAAAC	CGCCGAGGTCTTCTCCTGTCGCGATATAATGCGACAGG
....	
GTTTTTAGCCTACCTATAAGGAATTGAAAC	AGAGGAACAATTGGAATGTATGAAATAGGACAAAGA
GTTTTTAGCCTACCTAT	IS110 (1221bp)-IS110 (1221bp)
CTATAAGGAATTGAAAC	AGGTCAAACCTGGGGCAAAGACTTACGACGGGAAA
GTTTTTAGCCTACCTATAAGGAATTGAAAC	TGCCATCACCTATCATACTATCTCCATCCACTCTTA
....	
GTTTTTAGCCTACCTATAAGGAATTGAAAC	CACTTACGCGCATGCGGTAGCTCCTGATCATGTTCCG
GTTTTTAGCCTACCTATAAGGAATTGAAAC	MITE (309bp)
CTATAAGGAATTGAAAC	GTTTTTTGTACATTTCACTATCAAGGATTTTTCTAT
GTTTTTAGCCTACCTATAAGGAATTGAAAC	TGCCGTCTGTGCCTTGCGAATATTCTACGTCGCCGA
....	
GTTTTTAGCCTACCTATAAGGAATTGAAAC	CCGCCACATACATCCAAACAGGAAGTACCAGCATGG
GTTTTTAGCCTACCTAT	IS110 (1221bp)
CTATAAGGAATTGAAAC	AGAAGAATATTGACAGTTTAACTGCTCAAAAGATAGC
GTTTTTAGCCTACCTATAAGGAATTGAAAC	ACTGTCCCTCAATTTCCTTGTTTTCCTCCTTAAGCTT
....	
GTTTTTAGCCTACCTATAAGGAATTGAAAC	TGGATAGCAAAGGCAAGTGCAGAACTGGATAAAAAGA
GTTTTTAGCCTACCTAT	IS110 (1221bp)
CTATAAGGAATTGAAAC	CCTATCAGCTCCGGTGGCACCCCAAACGCAATAGC
GTTTTTAGCCTACCTATAAGGAATTGAAAC	GATAGCCTTCAGCTATTAACTTTGCCGCTTCCAATTG
....	
GTTTTTAGCCTACCTATAAGGAATTGAAAC	CCATGTTGGTTTTACGCTCAATCTCGGTTACGTAGAAA
GTTTTTAGCCTACCTAT	IS110 (1221bp)

Thermoanaerobacter pseudethanolicus ATCC 33223_ _NC_010321_121655_124879_48_our results

Thermoanaerobacter pseudethanolicus ATCC 33223_ _NC_010321_121655_124892_48_gold standard

Thermoanaerobacter pseudethanolicus ATCC 33223_ _NC_010321_126469_128012_23_our results

Thermoanaerobacter pseudethanolicus ATCC 33223_ _NC_010321_126525_128025_22_gold standard

Thermoanaerobacter pseudethanolicus ATCC 33223_ _NC_010321_131261_134422_47_our results

Thermoanaerobacter pseudethanolicus ATCC 33223_ _NC_010321_131313_134493_47_gold standard

Thermoanaerobacter pseudethanolicus ATCC 33223_NC_010321_134731_135597_13_our results

Thermoanaerobacter pseudethanolicus ATCC 33223_ _NC_010321_134784_135610_12_gold standard

Thermoanaerobacter pseudethanolicus ATCC 33223_ _NC_010321_137187_138123_14_our results

Thermoanaerobacter pseudethanolicus ATCC 33223_ _NC_010321_137241_138136_13_gold standard

Thermoanaerobacter pseudethanolicus ATCC 33223_ _NC_010321_139713_141450_26_our results

Thermoanaerobacter pseudethanolicus ATCC 33223_ _NC_010321_139765_141463_25_gold standard

GTCTTATCTGAACTATGAGGGATGTAAAC	ATAGAGGTACCAGGACAAGCTTGGCTCGAAGGAGAAG
....	
GTCTTATCTGAACTATGAGGGATGTAAAC	CTTTTCAGTTCAAATAATATCTCGTTCGCAAGGAAA
GTCTTATCTGAACTATGAGGGATGTAAAC	ATPase (792bp)-IS (1230bp)- IS (1071bp)
GTCTTATCTGAACTATGAGGGATGTAAAC	CTTTTTCTTTTTCAAAGACCTTGCACACAAAAAATC
....	
GTCTTATCTGAACTATGAGGGATGTAAAC	CATAGAAAGGCGGACTTTTTATTACTGGAAGGATGAAG
GTCTTATCTGAACTATGAG	IS (909bp)- IS(1230bp) –IS(438bp)
GAGGGATGTAAAC	ACAAAAAATTGCAAGGTTCTTTGGAACAAATATTGATGA
GTCTTATCTGAACTATGAGGGATGTAAAC	TCAACTTTTCGGTTTCTCTTTCAGCTTGTTTGACTGC
....	
GTCTTATCTGAACTATGAGGGATGTAAAC	ATTTCCAATGTTGCACATATAGGCAAATATCCGCTGCTA
GTCTTATCTGAACTATGAG	IS(1248bp)_IS(486bp)
....	IS(987bp)- IS(1230bp) –IS(321bp)
GAGGGATGTAAAC	ACATTGTTTGGGTCCTCCGATTTAGAGCCGATTGAGCCG
GTCTTATCTGAACTATGAGGGATGTAAAC	TTGCATAACTAGTGGGATCATTAATCGGCTCTATAATA
....	
GTCTTATCTGAACTATGAGGGATGTAAAC	AAGGGGGTGTTGGAAAAACTACTATTGCACTTGCAC
GTTTTATCTAAACTATGAGGGATGTAAAC	
....	IS (1131bp) –IS(1221bp)
GTTTACATCCCTCATAGTTCAGATAAGAC	TAAAGTTATTAATCCTCTTTCAGCCCACTTGTCGTA
....	
GTTTACATCCCTCATAGTTCAGATAAGAC	AGCTTTTTGGAACGACTAATCCAGATAGCCCTTATCA
GTTTACATCCCTC	IS110 (1287bp)
CTCATAGTTCAGATAAGAC	TATCAACATTGAACCAAAAGCTGTTATGTTGGTCATCA
GTTTACATCCCTCATAGTTCAGATAAGAC	TAACGCAGACACGGCAACAAAAGAAAAAGCGTTGATAA
....	
GTTTACATCCTTCATAGTTCAGATAAGAC	TGATGATTATTACCAACAACATACAGATATAAAAAA
GTTTACATCCTTCATAGTTCAGATAAGAC	

Caldicellulosiruptor saccharolyticus DSM 8903_NC_009437 _344075_347921_58 _our results

Caldicellulosiruptor saccharolyticus DSM 8903_NC_009437 _344075_347921_58 _gold standard

Caldicellulosiruptor saccharolyticus DSM 8903_NC_009437 _351757_353150_21 _our results

Caldicellulosiruptor saccharolyticus DSM 8903_NC_009437 _351757_353160_21 _gold standard

Caldicellulosiruptor saccharolyticus DSM 8903_NC_009437 _356437_357973_23 _our results

Caldicellulosiruptor saccharolyticus DSM 8903_NC_009437 _356504_357983_22 _gold standard

Caldicellulosiruptor saccharolyticus DSM 8903_NC_009437 _539664_541402_26 _our results

Caldicellulosiruptor saccharolyticus DSM 8903_NC_009437 _539731_541402_25 _gold standard

Caldicellulosiruptor saccharolyticus DSM 8903_NC_009437 _2513322_2520034_102 _our results

Caldicellulosiruptor saccharolyticus DSM 8903_NC_009437 _2513322_2519967_101 _gold standard

Caldicellulosiruptor saccharolyticus DSM 8903_NC_009437 _2522005_2524601_39 _our results

Caldicellulosiruptor saccharolyticus DSM 8903_NC_009437 _2521995_2524601_39 _gold standard

ATAGGTAGGCTAAAAAC	CAGCATTACCGCTTGTAAAAGTATTTGTATCTAATTTTAA
TTTCAATCCCTTATAGGTAAGCTAAAAAC	AAATTTTTGCAGGGGAGGAGACATAAAACGAAATGAA
………	
GTTTCAATCCCTTATAGGTAAGCTAAAAAC	CCACTCCCGCCCAAATTCAAAACCTCATTTTTTCCTT
GTTTCAATCCCTTATAGGTAAGCTAAAAAC	IS110 (1220bp)
ATAGGTAGGCTAAAAAC	TAGAGAGGGTAAAAGCGGCATGGCAAGTTTTTGAAA
GTTTCAATTCCTTATAGGTAGGCTAAAAAC	TTAAAGGAGGAAGATAAAAATGATAACTTCTGTAAA
………	
GTTTCAATTCCTTATAGGTAGGCTAAAAAC	GAAAACTAACCCGCAGCCGATGCGGGGAGATAAAAAAA
GTTTCAATTCCTTATAG	IS110 (1221bp)
ATAGGTAAGCTAAAAAC	AATGAAATTCAAATCTTCAACAGTAAAGATTTTGGC
GTTTCAATTCCTTATAGGTAGGCTAAAAAC	CACATACAGGACATTCCCACACCCGCTCCGATAACT
………	
GTTTCAATTCCTTATAGGTAGGCTAAAAAC	AACCAAACTTGAAAAAAGCTGCATTTTTCAAGTTTA
GTTTCAATTCCTTATAG	IS110 (1221bp)
ATAGGTAGGCTAAAAAC	CTTTTCTGACCACCAAAAACCTATGTGGCTGTTCTG
GTTTCAATTCCTTATAGGTAGGCTAAAAAC	GATTCTACTGCATTATTACATCTTGTGCTACATCA
………	
GTTTCAATTCCTTATAGGTAGGCTAAAAAC	TTTAAAATCGTATAAAGAGCTTATTGAGCTGTATTT
GTTTCAATTCCTTATAGGTAGGCTAAAAAC	

Thermoanaerobacter sp. X513_NC_014538_ _2149198_2149615_6_our results
Thermoanaerobacter sp. X513_NC_014538_ _2149254_2149615_5_gold standard

Thermoanaerobacter sp. X513_NC_014538_ _2340512_2341253_11_our results
Thermoanaerobacter sp. X513_NC_014538_ _2340499_2341198_10_gold standard

Thermoanaerobacter sp. X513_NC_014538_ _2342843_2347020_63_our results
Thermoanaerobacter sp. X513_NC_014538_ _2342830_2346967_62_gold standard

Thermoanaerobacter sp. X513_NC_014538_ _2348597_2362246_205_our results
Thermoanaerobacter sp. X513_NC_014538_ _2348597_2362246_205_gold standard

GTTTTTAGCTTACCTATAAGGGATTGAAAC	AAGGAAAAAATGAGGTTTTGAATTTGGGCGGGAGTGG
………	
GTTTTTAGCTTACCTATAAGGGATTGAAA	TTAAAATTAGATACAAATACTTTTACAAGCGGTAATGCTG
GTTTTTAGCCTACCTAT	IS110 (1220bp)
ATAGGTAGGCTAAAAAC	TAGAGAGGGTAAAAGCGGCATGGCAAGTTTTTGAAA
GTTTCAATTCCTTATAGGTAGGCTAAAAAC	TTAAAGGAGGAAGATAAAAATGATAACTTCTGTAAA
………	
GTTTCAATTCCTTATAGGTAGGCTAAAAAC	GAAAACTAACCCGCAGCCGATGCGGGGAGATAAAAAAA
GTTTCAATTCCTTATAG	IS110 (1221bp)
ATAGGTAAGCTAAAAAC	AATGAAATTCAAATCTTCAACAGTAAAGATTTTGGC
GTTTCAATTCCTTATAGGTAGGCTAAAAAC	CACATACAGGACATTCCCACACCCGCTCCGATAACT
………	
GTTTCAATTCCTTATAGGTAGGCTAAAAAC	AACCAAACTTGAAAAAAGCTGCATTTTTCAAGTTTA
GTTTCAATTCCTTATAG	IS110 (1221bp)
ATAGGTAGGCTAAAAAC	CTTTTCTGACCACCAAAAACCTATGTGGCTGTTCTG
GTTTCAATTCCTTATAGGTAGGCTAAAAAC	GATTCTACTGCATTATTACATCTTGTGCTACATCA
………	
GTTTCAATTCCTTATAGGTAGGCTAAAAAC	TTTAAAATCGTATAAAGAGCTTATTGAGCTGTATTT
GTTTCAATTCCTTATAGGTAGGCTAAAAAC	

Thermoanaerobacter sp. X514_ NC_010320_ _764967_765384_6 _our results
Thermoanaerobacter sp. X514 _NC_010320_ _764967_765328_5 _gold standard

Thermoanaerobacter sp. X514_ NC_010320_ _2340417_2341158_11 _our results
Thermoanaerobacter sp. X514_ NC_010320_ _2340404_2341103_10 _gold standard

Thermoanaerobacter sp. X514_ NC_010320_ _2342748_2346925_63 _our results
Thermoanaerobacter sp. X514_ NC_010320_ _2342735_2346872_62 _gold standard

Thermoanaerobacter sp. X514_ NC_010320_ _2348515_2363082_219 _our results
Thermoanaerobacter sp. X514_ NC_010320_ _2348502_2363082_219 _gold standard

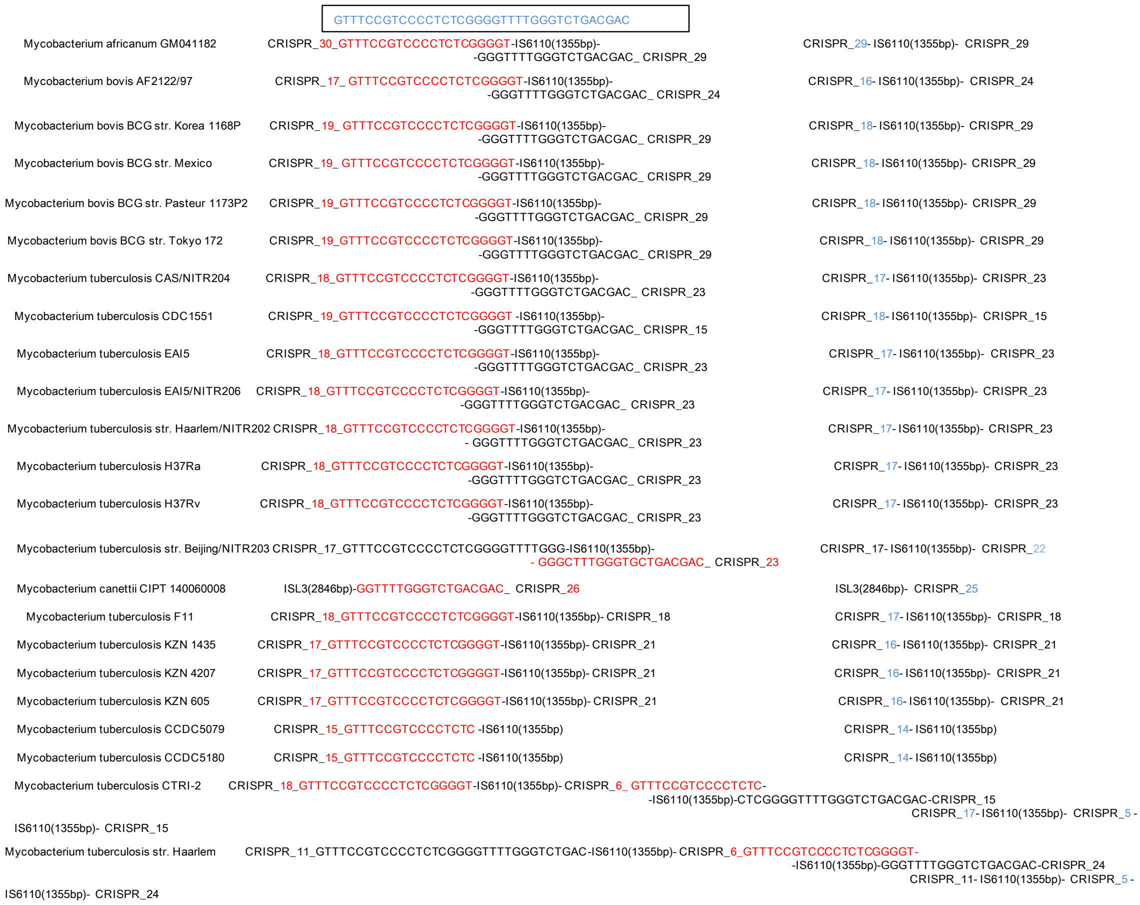

Figure S6. 15 CRISPRs broken by undetected DRs inside

(a)~(g) CRISPRs with undetected DRs inside. The two neighboring CRISPRs have almost identical DRs and one undetected DR in between. The undetected DR may be a full copy. The regions of new DRs matched to the existing DRs are in red. The regions of new spacers are marked by yellow background. The last number in the CRISPR name is the DR copy number.

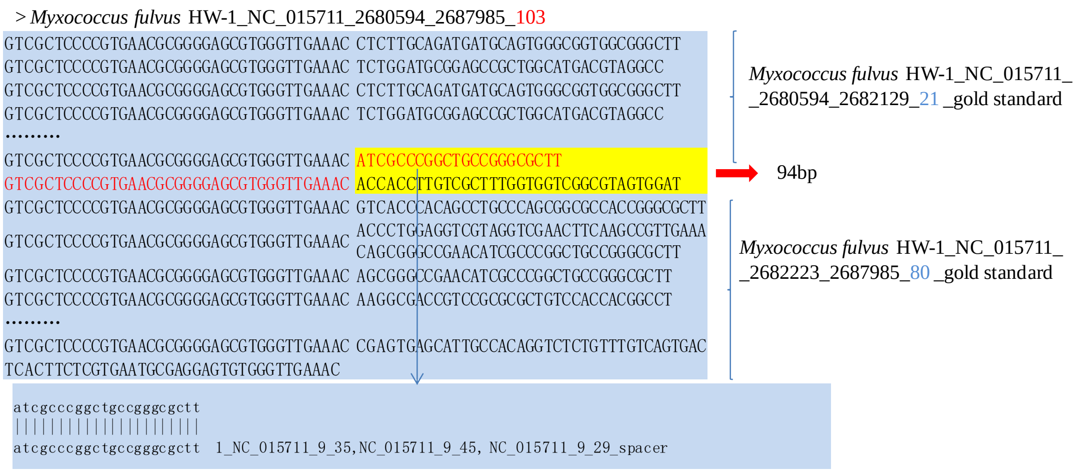

> *Caldicellulosiruptor obsidiansis* OB47_NC_014392_145343_158051_193

```
GTTTTATCTGAACTATGAGGGATGTAAAC  TCTTTTTTCTCTTTAACAAATCTTGATATGCCAACTCT
GTTTTATCTGAACTATGAGGGATGTAAAC  AACGACAAGCTAACGTACAGGAAAAACTCCCATGACG
GTTTTATCTGAACTATGAGGGATGTAAAC  GTTCAATTCTGATTATATCAGACTTAAAGTCCGTTG
GTTTTATCTGAACTATGAGGGATGTAAAC  TCAGTAGAATTTTTAAAAGATTATTTGACTTTAGGTGG
.........
GTTTTATCTGAACTATGAGGGATGTAAAC  TCAATGGCTAAAG
GTTTTATCTGAACTATGAGGGATGTAAAC  TCAAAATTTACAACAGACATTTTCTCTTCTTTCTTCTC
GTTTTATCTGAACTATGAGGGATGTAAAC  GCACAAAAACTGTGGACTACACTTTTTAAATCATTAAAG
GTTTTATCTGAACTATAAGGGATGTAAAC  GATACTATTCGAATGCACAATAAAAAACTTATTTTA
GTTTTATCTGAACTATGAGGGATGTAAAC  TTGAGAATAAGGATAATCTAACGAAGGGGTAACACCA
GTTTTATCTGAACTATGAGGGATGTAAAC  TCTCTACAGGAAATAATTTCATTAAATTATTCATAAA
.........
GTTTTATCTGAACTATGAGGGATGTAAAC  TTTATAGATGTATTTTTCATAGTACAGATATGCAACA
GTTTTATCTGAACTATGAG
```

Caldicellulosiruptor obsidiansis OB47_NC_014392_ _145343_157028_177_gold standard

80bp

Caldicellulosiruptor obsidiansis OB47_NC_014392_ _157108_158061_14 _gold standard

> *Thermosipho africanus* TCF52B_NC_011653_309784_311878_30

```
GTTTAGAATCTACCTATGAGGAATGAAAAC CATATTTTTTAGATAAAATATCACGCATTAAGTCCCCCA
GTTTAGAATCTACCTATGAGGAATGAAAAC TTGAATTCCTCGACACCCTCCTTTAAGATTTTCATTT
GTTTAGAATCTACCTATGAGGAATGAAAAC TATACTTCTCTATGTTGTCATTATAGTTATAGCAATATCTCGTG
GTTTAGAATCTACCTATGAGGAATGAAAAC ATTCAAATTCTTTCTTTAACATAATAACACCTCCATACATTT
.........
GTTTAGAATCTACCTATGAGGAATGAAAAC TAAATCAGT
GTTTAGAATCTACCTATGAGGAATGAAAAC ATCCCGATGTCCCGGTCTTCTCTACTATCTTGTATGGATTTT
GTTTAGAATCTACCTATGAGGAATGAAAAC TTCATTTTTGTATTATACATTTCAATTTCCTTTTTTAA
GTTTAGAATCTACCTATGAGGAATGAAAAC ATCACAGAATAATCTGTTGTCGATCGCCACTATTTTAAATC
GTTTAGAATCTACCTATGAGGAATGAAAAC GTGAATAAGTGTTTCTTCAAACTTTTCAAGACTAATGGTTTC
GTTTAGAATCTACCTATGAGGAATGAAAAC CAAAAATGGCACTTGCAACACTTTCTCCTTTTAGTTTCCTCA
.........
GTTTAGAATCTACCTATGAGGAATGAAAAC GTACGAAACTGTCTCCAATTTGCAACGGGGATAATTGTT
GTTTAGAATCTACCTATGAGGAATGAAA
```

Thermosipho africanus TCF52B_NC_011653_ _309784_310512_10 _gold standard

81bp

Thermosipho africanus TCF52B_NC_011653_ _310593_311880_18 _gold standard

> *Herpetosiphon aurantiacus* ATCC 23779_NC_009973_102032_109407_101

```
ATTTCAATTACTCGATCCGATTAGAGGATACTGAAAC  TACGGATGAGGCTGCACTCGCCGCTCAGGAGCCGCC
ATTTCAATTACTCGATCCGATTAGAGGATACTGAAAC  GTTGAAGGGATTCCATGTTTGATATTCACCCGCCGCT
ATTTCAATTACTCGATCCGATTAGAGGATACTGAAAC  ACGCATCGATCCGATCATGGATCGCCCACAACTGGT
ATTTCAATTACTCGATCCGATTAGAGGATACTGAAAC  AAACCTTTTATAGCTCGGCGCACTATGGCATGAT
.........
ATTTCAATTACTCGATCCGATTAGAGGATACTGAAAC  TCGCGCACTTGGCCAACCTTGGGCATTGGACAAACGT
ATTTCAATTACTCGATCCGAATTAGAGGATACTGAAAC TATCCAAATAACATTTGCCTATATTGCATCGCACATC
ATTTCAATTACTCGATCCGA TTAGAGGATACTGAAAC ATTCATCCATGGTTGCCAGAACCTTGGGGGCATAG
ATTTCAATTACTCGATCCGATTAGAGGATACTGAAAC  TCGATCACGTTGTACGGGCAACGCTTGCCATTAG
ATTTCAATTACTCGATCCGATTAGAGGATACTGAAAC  AGCAGCTTCATGGCGGCCTGCGGGTCGTGCAATTCG
ATTTCAATTACTCGATCCGATTAGAGGATACTGAAAC  GCCATGCCTCGGCTTGCTCACCAAATGGCTCCAT
.........
ATTTCAATTACTCGATCCGATTAGAGGATACTGAAAC  TGCATGGCGTTGAGTTCACGTGGCATCAAGCCAAGGCT
ATTTCAATTACTCGATCCGGCTGGACGAATTATTGA
```

Herpetosiphon aurantiacus ATCC 23779_NC_009973 _102032_108752_92 _gold standard

112bp

Herpetosiphon aurantiacus ATCC 23779_NC_009973 _108864_109408_7 _gold standard

> *Salmonella enterica* subsp. enterica serovar 4,[5],12:i:- str. 08-1736_NC_021820_4332392_4333865_24

```
  GTTTATCCCCGCTGGCGCGGGGAACA   TTTTTCAGCCCTTGTCGACTGCGGAACGCCCCT
CGGTTTATCCCCGCTGGCGCGGGGAACAC  GCGAAATAGTGGGGAAAAACCCCTGGTTAACC
CGGTTTATCCCCGCTGGCGCGGGAAACAC  TAGGCCTTGATACCATCGCTCGCACCTCGTCA
CGGTTTATCCCCGCTGGCGCGGGGAACAC  GTTTATTACTGCTTAGTTAATTAATGGGTTGC
.........
CGGTTTATCCCCGCTGGCGCGGGGAACAC  GATCGAGTAACGTGGCTGGAACGCG
                TCGGCGCGGGGAACAC  AAAATTAAAGCCGAGGGTGGCACCGCGCCTTATT
CGGTTTATCCCCGCTGGCGCGGGGAACAC  GCACCTCGAAACGGTTTTAAAACACTACCGTTT
CGGTTTATCCCCGCTGGCGCGGGGAACAC  TGGACCGATGGGGCCAACATCGCCGAACGTGG
CGGTTTATCCCCGCTGGCGCGGGGAACAC  GTTACGTTCGGTAAATGGAAAGCGGCGAATAT
CGGTTTATCCCCGCTGGCGCGGGGAACAC  CCAGAAAGTGCCGGTAGTGCCTGATGAACGAC
.........
CGGTTTATCCCCGCTGGCGCGGGGAACAC  CAGCACGAAAAATTATTTACTGTCGTTGCTCA
CGGTTTATCCCCGCTGGCGCGGGGAACAC
```

Salmonella enterica subsp. enterica serovar 4,[5],12:i:- str. 08-1736_NC_021820_4332390_4332968_9 _gold standard

75bp

Salmonella enterica subsp. enterica serovar 4,[5],12:i:- str. 08-1736_NC_021820_4333042_4333865_13 _gold standard

```
gatcgagtaacgtg-gctggaacgcg   Salmonella enterica subsp. enterica serovar Typhimurium str. 798
||||||||||||| ||||||||||||
gatcgagtaacgtgcgctggaacgcg   0.962_NC_017046_2_10_spacer
```

>*Salmonella enterica* subsp. enterica serovar Heidelberg str. 41578_NC_021810_6276_7936_27

>*Salmonella enterica* subsp. enterica serovar Heidelberg str. B182_NC_017623_3740803_3742463_27

>*Salmonella enterica* subsp. enterica serovar Heidelberg str. SL476_NC_011083_3051219_3052879_27

>*Salmonella enterica* subsp. enterica serovar Typhimurium str. 14028S_NC_016856_3096850_3098323_24

>*Salmonella enterica* subsp. enterica serovar Typhimurium str. D23580_NC_016854_3069600_3071012_23

>*Salmonella enterica* subsp. enterica serovar Heidelberg str. CFSAN002069_NC_021812_3668815_3670469_26

>*Salmonella enterica* subsp. enterica serovar Typhimurium var. 5_str. CFSAN001921-NC_021814_490784_492562_28

>*Salmonella typhimurium* LT2_NC_003197_3076613_3078147_25

> *Mycobacterium tuberculosis* RGTB423_NC_017528_3115972_3117672_23

```
              TCAGGGTTTTGGGTCTGACGAC TCGCGGCGAGCACGTCTCACCCAGCAGGCGGTGAGGTTGG
GTTTCCGTCCCCTCTCGGGGTTTTGGGTCTGACGAC ACGGACGAGCTGGACCGCATCAGCGATGCTGAGCTGAGG
GTTTCCGTCCCCTCTCGGGGTTTTGGGTCTGACGAC TTGTCTCAAGTCGTGCCGTCTGCGGTGACACGCTCCAA
GTTTCCGTCCCCTCTCGGGGTTTTGGGTCTGACGAC CTCGACGGCGCCAAGCCAGTTAGCGCAATCCA
.........
GTTTCCGTCCCCTCTCGGGGTTTTGGGTCTGACGAC ATGCGAATCCGCTGTCAGCACATGGGATCCGAGT
                                     CTAGGCGGCCCCCCGGCGAGGCTGGGGGCGGTTTCACGC
GTTTCCGTCCCCCG               TGACGAC GTTTCCGTCCGTCGT
GTTTCCGTCCCCTCTCGGGGTTTTGGGTCTGACGAC CAGCGCAGACGGCAGCCCCGAGTACTCGCTCTCCTCAG
GTTTCCGTCCCCTCTCGGGGTTTTGGGTCTGACGAC AGGCTGAAATTGAAGCCGGAAATGACGACGCATTGGT
GTTTCCGTCCCCTCTCGGGGTTTTGGGTCTGACGAC CTAAGCCCGCTAATCCCGCACAAGTGGTCAGAAAA
GTTTCCGTCCCCTCTCGGGGTTTTTGGGTCTGACGACCTGATGATTGGTCGGCGTATGACGTGCTACTGAGGTGTT
.........
GTTTCCGTCCCCTCTCGGGGTTTTGGGTCTGACGAC GTGCAAGAATTCCGGGTTGCAGTGCAACACGGTTTTAA
GTTTCCGTCCCCTCTCGGGGTTTTGGGTCTGACGAC
```

Mycobacterium tuberculosis RGTB423_NC_017528 _3115959_3116942_13_gold standard

109bp

Mycobacterium tuberculosis RGTB423_NC_017528 _3117050_3117672_8_gold standard

```
atgcgaatccgctgtcagcacatggga-tccgagt Mycobacterium tuberculosis H37Rv
||||||||||||||||||||||||||| |||||||
atgcgaatccgctgtcagcacatgggattccgagt  97.1%_NC_000962_6_14_spacer
```

```
ctaggcggcccccggcgaggctgggggcggtttcacgc Mycobacterium tuberculosis H37Rv
|||||||||||  |||||||||||||||||||||||||
ctaggcggcccc--ggcgaggctgggggcggtttcacgc   94.9%_NC_000962_6-15 spacer
```

> *Thermococcus onnurineus* NA1_NC_011529_994458_997976_52

```
TTTCGATTCTCTTAGAATCTTATTGCAAC       TAAGAAATGGGGCACTAGCTTCGGCGGTTGGAATTTG
GTTTCAATTCTCTTAGAGTCTTATTGCAAC      CTGTGGGTGGTTCCACCAGAGCTTTGCAACGATTAC
TTTCAATTCTCTTAGAGTCTTATTGCAAC       CCTTGAAATCCCGATAAGCGGGAGCTACGAGTTTC
GTTTCAATTCTCTTAGAGTCTTATTGCAAC      TTCTTGCCACGGTCGCCTACATCACAAGCTACGATGA
.........
GTTTCAATTCTCTTAGAGTCTTATTGCAAC      TCGCTTCGACGGTGGCTTTTGTCTCCGGATTCATCATC
GTTTCAATTCTCTTA          TTGAGAC      GAAGTTGGCAACTCCAACACATGCGCCC
GTTTCAATTCTCTTAGAGTCTTATTGCAAC      CAGATCGCCTGCTGGGTCTGGCTCATCTGGCCGTGCTCGC
TTTCAATTCTCTTAGAGTCTTATTGCAAC       CCGGCATCAGGGCTGACACCTACTCAACGACCCTCTATG
GTTTCAATTCTCTTAGAGTCTTATTGCAAC      AGGAGGAAGATGGCAAGTATCATGTTTATGATAAGAA
GTTTCAATTCTCTTAGAGTCTTATTGCAAC      ACGAATCTATCAACCGAGTCAATGGCCCGAGTTCTA
.........
GTTTCAATTCTCTTAGAGTCTTATTGCAAC      TCCCGTTCACGAGCTGTTGGCCGGCCTGCGCAATCTG
GTTTCAATTCTCTTAGAGTCTTATTGCAAC
```

Thermococcus onnurineus NA1_NC_011529 _994457_994969_7 _gold standard

88bp

Thermococcus onnurineus NA1_NC_011529 _995057_997976_43_gold standard

Figure S7. 8 CRISPRs broken at the beginning of circular chromosomes

(a) A CRISPR is broken into two at the beginning of a circular chromosome of *Cyanobacterium aponinum* PCC 10605. One more spacer is proposed to combine the two CRISPRs into one longer CRISPR. The added region is highlighted in red. The last number in the CRISPR name is the DR copy number.

(b)~(h) The other CRISPRs are broken into two at the beginning of a circular chromosome.

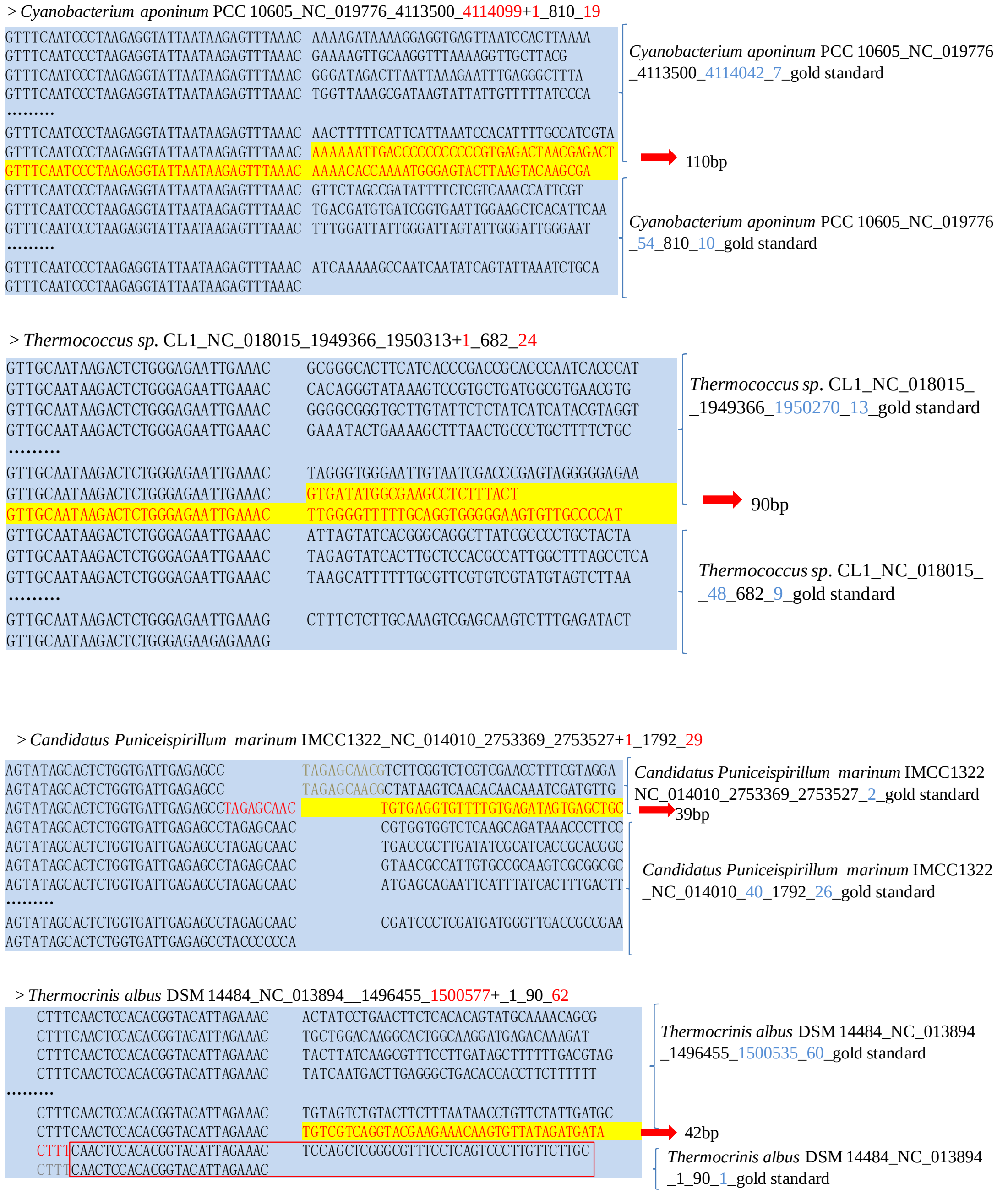

> *Fervidicoccus fontis* Kam940_NC_017461_ 1319183_1319206 +1_794_13

```
GAATCTCTCAGATAGAATTGAAAG    TCTGAAAAAATCATCACAACAATCAGCTTTTCCCT
GAATCTCTCAGATAGAATTGAAAG    TTGGCATATTTACTCCCCTTGCTCAGAATCAAGCATT
GAATCTCTCAGATAGAATTGAAAG    TTGGCATATTTACTCCCCTTGCTCAGAATCAAGCATT
GAATCTCTCAGATAGAATTGAAAG    TTCACAAATTTAGTCACAAATGCGCTAGGAAACAGAA
GAATCTCTCAGATAGAATTGAAAG    TCAAACCCATGCTTTCTTAGTATTTCTGCTGCTTCT
GAATCTCTCAGATAGAATTGAAAG    CTCTTGCTAATGCTCTAGCAGTCGATCATAGTAGGC
………
GAATCTCTCAGATAGAATTGAAAG    AACTCTTCCATGTATTCAACCAAACTTTCCAAATCCT
GAATCTCTCAGATAGAATTGAAAG
```

60bp

Fervidicoccus fontis Kam940_NC_017461 _37_794_12 _gold standard

> *Picrophilus torridus* DSM 9790_NC_005877_1540318_1545895+1_309_87

```
CTTTCAATCCTATTTAGGTTGTTATTTAAC  TCCGCCGAATTTCAAACACTTATAGATGGACTAGA
TTTTCAATCCTATTTAGGTTATTATTTAAC  TATCTCTCTTTTCATTCCTTATCGATTTATCGATCC
CTTTCAATCCTATTTAGGTTATTATTTAAC  CACGGTGGTGATGCAACGATAAGATCAACATTATCA
CTTTCAATCCTATTTAGGTTATTATTTAAC  TCCATCTAAAGACATTCTCTCTAAATATATCAAAGCC
………
CTTTCAATCCTATTTAGGTTATTATTTAAC  TATAATCCTGCTTCCCTACATATCTAACAGAACCTCT
CTTTCAATCCTATTTAGGTTATTATTTAAC  CATTGATTTTTCATTGAAATTCATGAATTCGGATAA
CTTTCAATCCTATTTAGGTTATTATTTAAC  CTGGATTTCCTTGGCCATATTTGCCGAGATACTATA
CTTTCAATCCTATTTAGGTTATTATTTAAC  ATATATCGGATTTCAAACCCTTATAGATAGACTAGA
CTTTCAATCCTATTTAGGTTATTATTTAAC  GCCAGAGGCATTTCTCCGTGCGTGGTCCTTTAATGA
CTTTCAATCCTATTTAGGTTATTATTTAAC  ACAACAGGCAAAAACAGAATTAAGACTGGCCATTGAG
CTTTCAATCCTATTTAGGTTATTATTTAAC
```

Picrophilus torridus DSM 9790_NC_005877 _1540318_1545873_82 _gold standard

36bp

Picrophilus torridus DSM 9790_NC_005877 _15_309_4 _gold standard

> *Thermococcus litoralis* DSM 5473_NC_022084_2214800_2215172+1_2779_46

```
CTTTCAATTCTTTTAAAGTCTTATTGGAAC    AAAGCGTTATTAAGATGTAGTGCCCTACTCTCCAT
CTTTCAATTCTTTTAAAGTCTTATTGGAAC    CTGTTTCATCCTACGCGCGACAAGAATTGGATGATC
CTTTCAATTCTTTTAAAGTCTTATTGGAAC    ATAGGAGTGTCTTCCCAGTCAATCTTCTTCTGGTATGT
CTTTCAATTCTTTTAAAGTCTTATTGGAAC    ATATATATAATTAATTCGTTTACTGTACAATAAATTA
CTTTCAATTCTTCTAAAGTCTTATTGGAAC    CACCTCAGAATATGTTGAAGTTTAGGCCTGTGATGCT
CTTTCAATTCTTCTAAAGTCTTATTGGAAC    ACCGAAAAGAAGAACCAAAAGGAGGAGGTGAAAATAGA
CTTTCAATTCTTCTAAAGTCTTATTGGAAC    AGCTTGATGGCTTTGAATGAAAAAACTGCGCTCTTGA
CTTTCAATTCTTCTAAAGTCTTATTGGAAC    TAAACTTGCTTGAGATTTCGCGGCGATTTTACTATA
CTTTCAATTCTTCTAAAGTCTTATTGGAAC    AAAGACAACTCGTAACAATTGGGTTGGTCTTGCTGTT
CTTTCAATTCTTCTAAAGTCTTATTGGAAC    TTGAAAAAGAACTCAGGGAGCAATCAACCTATACAACGA
CTTTCAATTCTTCTAAAGTCTTATTGGAAC    AGCTTGATGGCTTTGAATGAAAAAACTGCGCTCTTGA
………
CTTTCAATTCTTCTAAAGTCTTATTGGAAC    GATGAGAACGGGACGAAGCTAAGTTACTACGTGGAGAGT
CTTTCAATTCTTCTAAAGTCTTATTGGAAC
```

Thermococcus litoralis DSM 5473_NC_022084 _2214800_2215162_5_gold standard

38bp

Thermococcus litoralis DSM 5473NC_022084 _29_2779_40 _gold standard

> *Acidilobus saccharovorans* 345-15_NC_014374_1495994-1496453+1-1050_23

```
GTTTCAATAAAGTTTCACGGTTTA  ACTTCTGGGAGTGAGGCACCTGGATAGCCGCCACAGC
TTTTCAGCGCCATCTTGTGGTTTC  TTCGAGGATAGGGAGAATGCCAAAATATGGACAGAGGTGTTTACGCTGTCTATGAA
GTTTCAACACCATCAGATGGTTTC  CTCCACGTCTACGGCAATGAACCTGAACAGCAACGCAACGC
GTTTCAACACCATCAGATGGTTTC  AGAAATCAGTAGTCCCAGGTATAGACGTCCCAAACAGATA
………
GTTTCAACACCATTCCTTGGTTTC  ACTGCCTTCAGTGCCACGGTTGGCGGCATAAACACTG
GTTTCAACACCATTCCTTGGTTTC  CTGGAGAGGCCCCACAGGCCTCCCCTGAGGCCCAGACATCCA
GTTTCAACACCATTCCTTGGTTTC  CCATTGGTAAATGAGATTGTGGTTACGCATCCAGAGCATAA
GTTTCAACACCATTCCTTGGTTTC  GCCGTTGAAGTAGTCCTCGTCGTCGTGCACCACCTCCTCCA
GTTTCAACACCATTCCTTGGTTTC  TGATTGTAACTGGGGCGCTGACAGGGTACATGTAGGTG
GTTTCAACACCATTCCTTGGTTTC  ACGTGCCCTATGGAACGCTGAAGGTATACCTATCAAGGTCAA
………
GTTTCAACACCATTCCTTGGTTTC  TCGGAAAGAGAGGAAAGAAGGGAAAGGGGTAACGTTGTTAA
GTTTCAACACCATTCCTTGGTTTC
```

Acidilobus saccharovorans 345-15_NC_014374 _1495994_1496411_6_gold standard

42bp

Acidilobus saccharovorans 345-15_NC_014374 _1_1050_16_gold standard
